# Ankyrin-3 as a molecular marker of early-life stress and vulnerability to psychiatric disorders

**DOI:** 10.1038/tp.2016.211

**Published:** 2016-11-08

**Authors:** A Luoni, R Massart, V Nieratschker, Z Nemoda, G Blasi, M Gilles, S H Witt, M J Suderman, S J Suomi, A Porcelli, G Rizzo, L Fazio, S Torretta, A Rampino, A Berry, P Gass, F Cirulli, M Rietschel, A Bertolino, M Deuschle, M Szyf, M A Riva

**Affiliations:** 1 Department of Pharmacological and Biomolecular Sciences, University of Milan, Milan, Italy; 2 Department of Pharmacology and Therapeutics, McGill University, Montreal, QC, Canada; 3 Department of Genetic Epidemiology in Psychiatry, Central Institute of Mental Health, Medical Faculty Mannheim/Heidelberg University, Mannheim, Germany; 4 Department of Psychiatry and Psychotherapy, University of Tuebingen, Tuebingen, Germany; 5 Sackler Institute for Epigenetics and Psychobiology, McGill University, Montreal, QC, Canada; 6 Department of Basic Medical Science, Neuroscience and Sense Organs, University of Bari Aldo Moro, Bari, Italy; 7 Department of Psychiatry and Psychotherapy, Central Institute of Mental Health, Medical Faculty Mannheim/Heidelberg University, Mannheim, Germany; 8 McGill Centre for Bioinformatics, McGill University, Montreal, QC, Canada; 9 Laboratory of Comparative Ethology, Eunice Kennedy Shriver National Institute of Child Health and Human Development, National Institutes of Health, Bethesda, MD, USA; 10 Department of Cell Biology and Neurosciences, Istituto Superiore di Sanità, Rome, Italy

## Abstract

Exposure to early-life stress (ELS) may heighten the risk for psychopathology at adulthood. Here, in order to identify common genes that may keep the memory of ELS through changes in their methylation status, we intersected methylome analyses performed in different tissues and time points in rats, non-human primates and humans, all characterized by ELS. We identified *Ankyrin-3* (*Ank3*), a scaffolding protein with a strong genetic association for psychiatric disorders, as a gene persistently affected by stress exposure. In rats, *Ank3* methylation and mRNA changes displayed a specific temporal profile during the postnatal development. Moreover, exposure to prenatal stress altered the interaction of ankyrin-G, the protein encoded by *Ank3* enriched in the post-synaptic compartment, with PSD95. Notably, to model in humans a gene by early stress interplay on brain phenotypes during cognitive performance, we demonstrated an interaction between functional variation in *Ank3* gene and obstetric complications on working memory in healthy adult subjects. Our data suggest that alterations of *Ank3* expression and function may contribute to the effects of ELS on the development of psychiatric disorders.

## Introduction

Adverse events during prenatal and early postnatal periods have a pivotal role in the later susceptibility to neuropsychiatric disorders^1, 2, 3^ by interfering with the developmental trajectories of different systems, thus leading to long-lasting reprogramming consequences.^4^ A growing body of evidence in the last decade has ascribed a key role to the epigenome, an array of chemical modifications to the DNA and histone proteins that affect gene expression without altering the DNA sequence, in bridging the experience of early insults with the appearance of a pathological phenotype at adulthood.^5, 6, 7^ In particular, several rodent and human studies have proven that among epigenetic mechanisms, DNA methylation is dynamically sensitive to external cues particularly in the perinatal period, when most of the DNA methylation patterns are arranged to shape and define the cellular destiny and thus are also highly responsive and susceptible to environmental stressors that could alter such programming, thus increasing the vulnerability to later psychopathology.^8, 9, 10, 11, 12, 13^ In order to investigate the underlying causative mechanisms responsible of the long-term effects, several animal models have been developed that replicate the exposure to different types of stressors, of diverse intensities, in specific time windows during early perinatal life.^14, 15, 16, 17^ Although these animal models bear the advantages of studying, in few months, the effects of neonatal stress on adult behavior, both in the brain as well as at peripheral level, they have the limitation of lacking a direct relevance to the human condition. Here, in order to circumvent these problems, we used a cross-species genome-wide approach to identify shared DNA methylation patterns that are associated with early-life stress (ELS). In detail, we analyzed: (1) in humans the methylome signature characterizing CD34^+^ cells derived from the cord blood of newborns whose mothers have been characterized for stressful experiences during the last trimester of gestation;^18^ (2) in rhesus monkeys (*Macaca mulatta*) the DNA methylation patterns in the peripheral blood and in the prefrontal cortex (PFC) of monkeys that were exposed to different early-life social and rearing conditions;^19, 20^ (3) in rodents (*Rattus norvegicus*) we addressed the PFC of early-adult rats exposed to prenatal stress (PNS), a well-established model of vulnerability to altered adult behavior.^18, 21, 22, 23, 24^

This combined analysis pointed to *Ankyrin-3* (*Ank3*), a genetic risk factor for bipolar disorder and schizophrenia ^25, 26, 27^ that plays a pivotal role as scaffolding protein in specific membrane domains, such as the node of Ranvier and the axon initial segment.^28, 29, 30^ Moreover, a role for ankyrin-G (ANKG) protein in regulating the post-synaptic compartment organization and function has recently emerged,^31, 32^ suggesting its potential for mediating the effects of stress exposure on dysfunctions associated with several psychiatric conditions.^33, 34, 35, 36^

Finally, we demonstrated, in healthy adult subjects, that variation in the *Ank3* gene affecting gene expression interacted with an early stress factor (obstetric complications), in modulating prefrontostriatal connectivity and behavioral correlates of working memory, a cognitive phenotype tightly linked with different psychiatric disorders.^37^ Overall, our results suggest that alterations of *Ank3* expression may contribute to the effects of perinatal stress on the vulnerability to psychiatric conditions.

## Materials and Methods

### Human cohort for MeDIP-chip analysis

Data were obtained from a cohort of mothers and their infants (*n*=180) recruited during the third trimester of pregnancy in the Rhine-Neckar Region of Germany. For inclusion and exclusion criteria see Supplementary Information. The study protocol was approved by the Ethics Committee of the Medical Faculty Mannheim of the University of Heidelberg. The study was conducted in accordance with the Declaration of Helsinki. All mothers provided written informed consent before participation. Investigator was blinded for the laboratory analyses.

### Animals and experimental paradigms

Rhesus monkeys were reared as previously described.^18, 19^ Venous blood samples were obtained from 30 days old and 2-year-old monkeys, whole blood and buccal epithelial cell samples were taken at 2-years or older age, while PFCs were obtained from 7-year-old male monkeys. The Institutional Animal Care and Use Committee of the NICHD approved protocols for the use of experimental animals.

Pregnant rats were randomly assigned to control (Ctrl, *n*=7) or PNS (*n*=9) conditions. The stress paradigm was carried out as previously described.^18, 38, 39^ Male offspring PFC was dissected at different postnatal days (PND: 7 (Ctrl, *n*=7; PNS, *n*=9), 21 (Ctrl, *n*=6; PNS, *n*=6) and 62 (Ctrl, *n*=7; PNS, *n*=9)). Rat handling and experimental procedures were performed in accordance with the EC guidelines (EC Council Directive 86/609 1987) and with the Italian legislation on animal experimentation (D.L. 116/92), in accordance with the National Institute of Health Guide for the Care and Use of Laboratory Animals.

All efforts were made to minimize animal suffering and to reduce the number of animals used, which has been set, using G*Power 3 software,^40^ to take into account mortality and to allow *n*=7–9 in each final group, also based upon our own previous data.^39^ No pre-established inclusion/exclusion criteria were used for subsequent analyses. All samples were processed and analyzed by experimenters blind to rearing or prenatal stress conditions.

### Extraction of DNA

Genomic DNA was extracted using Qiagen (Hilden, Germany) or Promega (Madison, WI, USA) systems, sheared by sonication and quantified using the Qubit system (Life Technologies, Burlington, ON, Canada).

### Analysis of genome-wide promoter DNA methylation

MeDIP analysis was adapted from previously published protocols.^18, 19^ In detail, the final sample size for the methylome analysis was the following: rat PFC, Ctrl *N*=4, PNS *N*=4; human cohort, Ctrl *N*=8, ELS *N*=10; monkey PFC, Ctrl *N*=4, ELS *N*=4; monkey whole blood, Ctrl *N*=5, ELS *N*=5; monkey buccal samples, Ctrl *N*=3, ELS *N*=3; monkey CD3^+^ (30 days old), Ctrl *N*=10, ELS *N*=10; monkey CD3^+^ (2 years old), Ctrl *N*=6, ELS *N*=4 (the monkey CD3^+^ samples were pooled and subjected to 3 parallel MeDIP analyses, that is 3 Ctrl and 3 ELS pools per time point). Briefly, 2 μg of DNA were sonicated, and methylated DNA was immunoprecipitated using anti-5-methyl-cytosine (Cat. No. BI-MECY-0100, Eurogentec, Fremont, CA, USA). The DNA–antibody complex was immunoprecipitated with protein G, and the methylated DNA was re-suspended in digestion buffer (50 mm TrisHCl pH8; 10 mm EDTA; 0.5 % SDS) and treated with proteinase K overnight at 55 °C. The input and bound fractions were purified, amplified using the Whole Genome Amplification Kit (Sigma-Aldrich, St. Louis, MO, USA), and labeled for microarray hybridization with Cy3-dUTP and Cy5-dUTP, respectively, using the CGH Enzymatic Labeling Kit (Agilent Technologies, Mississauga, ON, Canada) in accordance with the manufacturer's instructions. Custom designed tiling arrays were used (Agilent Technologies). All steps of the hybridization, washing, scanning and feature extraction procedures were performed in accordance with the Agilent Technologies protocol for chip-on-chip analysis. Extracted microarray intensities were processed and analyzed using the R software environment for statistical computing (http://www.r-project.org/). The data discussed in this publication have been deposited in NCBI's Gene Expression Omnibus^41^ and are accessible through GEO series accession numbers GSE84028 (subseries accession numbers: GSE84018, GSE84020, GSE84021 and GSE84024).

### Validation using qPCR

Gene-specific validation of MeDIP data was performed applying quantitative-real-time PCR (qPCR) (see Supplementary Table 2) using the 2^−ΔΔCt^ method. Data are expressed as group means±s.e.m. To test for statistical significance, the Student's *t*-test was used (two-tailed), and the alpha level was set at 0.05.

### Gene expression analysis in rats

RNA was isolated from rat PFC using AllPrep DNA/RNA Mini kit (Qiagen). RNA was analyzed by TaqMan qRT-PCR instrument (CFX384 real-time system, Bio-Rad Laboratories, Segrate, Italy) using the iScriptTM one-step RT-PCR kit for probes (Bio-Rad Laboratories) in triplicate as multiplexed reactions with a normalizing internal control (*36b4*).

### Protein analysis

Protein subcellular fractionating was obtained homogenizing the tissues in a Teflon-glass potter in ice-cold 0.32 m sucrose buffer containing 1 mm HEPES, 1 mm MgCl_2_, 1 mm NaHCO_3_ and 0.1 mm phenylmethylsulfonyl fluoride, pH=7.4, in the presence of a complete set of protease (Roche, Monza, Italy) and phosphatase (Sigma-Aldrich) inhibitors. Total cell homogenate was next processed to obtain the crude membrane fraction (P2) and the Triton-Insoluble post-synaptic fraction (TIF) as described in detail in the Supplementary S1 Materials and Methods.

For western blot analysis, samples were run on SDS-polyacrylamide gel electrophoresis (PAGE) and analyzed using the Chemidoc MP Imaging System after normalization on β-actin levels.

Co-immunoprecipitation assays were performed as in Vastagh *et al.*^42^ with the introduction of some methodological modifications. Briefly, total cell homogenates from the rat PFC were immunoprecipitated with 3 μg of antibody overnight at 4 °C, followed by a 2 h incubation with protein A/G beads. Beads were then washed extensively and bound complexes were analyzed by SDS-PAGE and western blotting.

For all the above-mentioned molecular and biochemical analyses, all data met the assumptions of normal distribution and equality of variance.

### Interaction between functional variation in Ank3 and obstetric complications on working memory processing

#### Association of rs9804190 with human post-mortem prefrontal Ank3 mRNA expression

Earlier results have indicated association of an intronic single-nucleotide polymorphism in the *Ank3* gene, that is rs9804190, with gene expression levels in the superior temporal gyrus of patients with schizophrenia.^28^ We therefore tested such association in a large group of samples from PFC (BA46) of 268 non-psychiatric individuals using Braincloud (http://braincloud.jhmi.edu/) (Supplementary Information and Supplementary Table 3). In particular, analysis of covariance was performed, with rs9804190 genotype as the independent variable, *Ank3* mRNA expression as the dependent variable, and non-matched variables between groups (that is, age, sex, RNA integrity number) and ethnicity as covariates of no interest. A statistical threshold of *P*<0.05 was used for this analysis.

### *In vivo* fMRI and behavioral study

Three hundred six healthy adults (Supplementary Information and Supplementary Table 4) were enrolled in a behavioral study. One hundred seventy four of these individuals (supplementary Table 4) also participated in a functional magnetic resonance imaging (fMRI) study. In both studies, all individuals performed the 1- and 2-back versions of the N-back task, eliciting two loads of working memory (WM) processing and were genotyped for rs9804190 (Supplementary S1 Materials and Methods). Furthermore, mothers of all individuals completed the McNeil-Sjöström Scale,^43^ which allowed to split individuals based on the Obstetric Complications (OC) score (Supplementary S1 Materials and Methods).^44^ Sample sizes were: (a) in the fMRI sample, 57 CC with OC, 48 CC without OC, 38 T carriers with OC, 31 T carriers without OC; (b) in the behavioral study sample, 115 CC with OC, 66 CC without OC, 86 T carriers with OC, 39 T carriers without OC.

### fMRI data acquisition and analysis

fMRI data were acquired with a 3T GE scanner and processed with SPM8 (see Supplementary S1 Materials and Methods). Second-level random effects multiple regression were performed to investigate *Ank3* × OC interaction on prefrontal activity during performance of the 1- and 2-back WM tasks, using task load as the repeated-measures factor, and *Ank3* rs9804190 genotype as well as OC (absence/presence) as the between-subjects factor. We used a statistical threshold of *P*<0.05, family-wise error small volume corrected for the left dorsolateral PFC (DLPFC), whose role is crucial in WM processing^45^ and the greater involvement of the left portion of DLPFC during WM tasks eliciting both verbal and visuospatial processing.^46, 47^ Then, we explored the interaction between *ANK3* and OC on prefrontostriatal connectivity. With this aim, psychophysiological interaction analysis ^48^ was performed for each subject. In particular, we used a 5 mm region of interest centered on the peak activity (*x*=−48, *y*=38, *z*=30) in left DLPFC as seed region (Supplementary S1 Materials and Methods). Individual psychophysiological interaction contrasts were then entered in second-level random effects multiple regressions using task load as the repeated-measures factor, and *Ank3* rs9804190 genotype as well as OC (absence/presence) as the between-subjects factors. We used a statistical threshold of *P*<0.05, family-wise error small-volume corrected within the left striatum (see Supplementary S1 Materials and Methods).

### Analysis of behavioral data

A repeated measure factorial analysis of variance was performed, with genotype and OC presence/absence as the between-subjects factors, load (1- and 2-back) as the repeated-measures factor and behavioral accuracy (% of correct responses) or reaction time as the dependent variable. Tukey's test was used for *post hoc* analyses.

## Results

### Genome-wide DNA methylation analyses in different paradigms of ELS exposure

MeDIP-chip analysis at PND62 in the PFC of male rats uncovered 7660 probes associated with 3475 distinct genes whose methylation status was different between control (Ctrl) and animals exposed to PNS (false discovery rate (FDR)<0.2). In particular, 3362 probes, related to 1508 genes, were hypomethylated in PNS animals, whereas 4298 probes, corresponding to 1773 genes, were hypermethylated in PNS animals. Furthermore, the remaining 194 genes showed mixed methylation patterns. Using Ingenuity Pathway Analysis, we found enrichment for several neuronal functions, including ‘psychological disorders' (Supplementary Table 5), among other important biological mechanisms.

**Figure 1:**
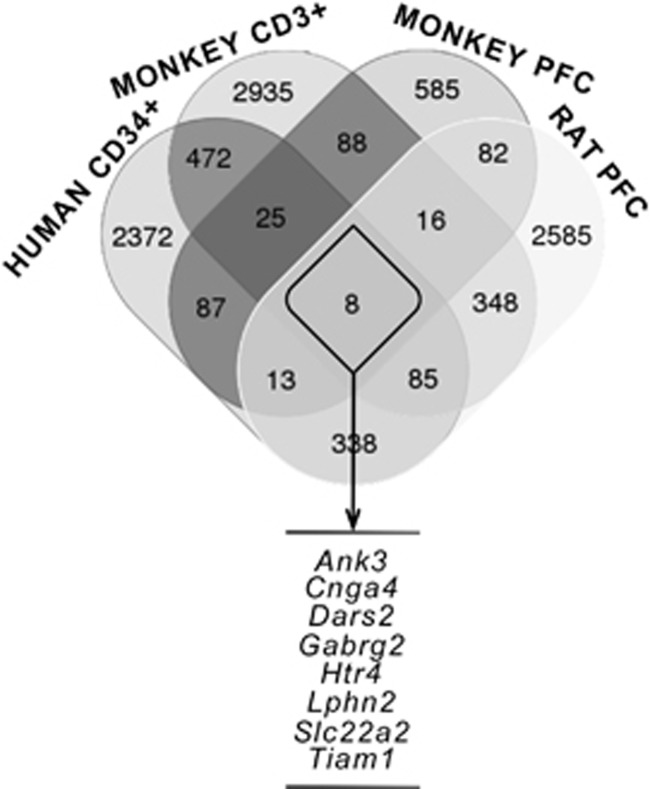
Venn diagram showing the overlap of the number of differentially methylated genes regulated by exposure to early-life stress in different species (see Materials and Methods for details). The eight genes listed below emerged as differentially methylated after early-life stress in all conditions. PFC, prefrontal cortex.

In order to prioritize the genes that may be important in keeping the memory of the adverse perinatal experience through the regulation of their methylation status, we performed a cross-species analysis overlapping the list of genes differentially methylated in the PFC of rats exposed to PNS with the results of MeDIP-chip genome-wide analyses in two different species, humans and non-human primates, which were also characterized by exposure to adversities during the perinatal life. In the human cohort, we analyzed the methylation status of genes in CD34^+^ hematopoietic stem cells collected from the umbilical cord of newborns from mothers with different levels of stress during gestation.^18^ In addition, we used two groups of monkeys characterized by different early-life social and rearing conditions, in which we performed genome-wide analyses in the PFC of 7-year-old animals and in the peripheral T cells (CD3^+^) at ages 30 days and 2 years, in order to look for stable peripheral DNA signatures in response to different early environmental conditions.^49, 50^ The analyses in monkeys, in particular, at a difference from our previously published work,^18^ fill a gap as the addition of the data from the monkey PFC further integrates the results obtained at brain level (in adult rats and monkeys) with data obtained at peripheral level (in monkeys and human newborns), providing more reliability and authenticity to our trans-species approach. We identified eight genes commonly affected—regardless the direction of the methylation changes—after exposure to ELS in all the conditions described above: *Ankyrin-3* (*Ank3*), *Cyclic nucleotide gated channel alpha 4* (*Cnga4*), *Aspartyl-tRNA synthetase 2, mitochondrial* (*Dars2*), *GABA A receptor, gamma 2* (*Gabrg2*), *5-hydroxytryptamine receptor 4* (*Htr4*), *Latrophilin 2* (*Lphn2*), *Solute carrier family 22, member 2* (*Slc22a2*) and *T-cell lymphoma invasion and metastasis 1* (*Tiam1*; Figure 1). Almost all these genes showed a mixed direction of the methylation changes, with the exception of *Ank3* and *Gabrg2*, which showed a consistently higher methylation in the stressed group across all the conditions (Supplementary Table 6). *Ank3* has previously emerged as a common genetic risk factor for neurodevelopmental and psychiatric disorders, such as bipolar disorder^26, 27^ and schizophrenia,^28^ whereas a strong dysfunction of the GABAergic system has also been demonstrated in anxiety and depression.^51, 52^ We focused our subsequent analyses on *Ank3* since its methylation status was affected in the same direction also in the whole blood and buccal cells from 2-year-old monkeys exposed to ELS, whereas *Gabrg2* did not (Supplementary Table 7). The extent of the methylation differences pertaining all the *Ank3* probes hypermethylated in the stressed groups is listed in Supplementary Table 8 using similar significance (*P-*values) and effect size estimates (as differential expressed in log2) as previously reported.^18, 19^

### Validation of MeDIP-chip

**Figure 2:**
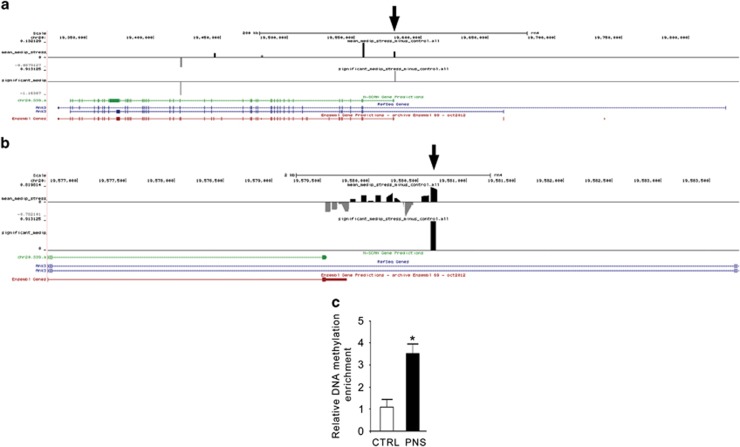
Expanded views from the UCSC genome browser at the rat Ank3 gene location are depicted. (**a**) The first track shows average methylation probe fold differences (Log2), whereas the second track shows the regions whose methylation status is significantly different as a consequence of PNS. The three tracks at the bottom show exons and introns boundaries taken from the rat N-SCAN Gene Predictions, NCBI Reference Sequence Database (RefSeq) and Ensembl Gene Predictions, respectively. Arrow indicates the location of DNA amplification for qPCR validation. (**b**) Zoomed view of **a** showing every single probe in the region selected for the validation in the rat prenatal stress model. (**c**) Bar graph of the qPCR validation of *Ank3* relative DNA methylation enrichment between PNS and Ctrl groups, shown as relative bound fraction concentrations. The data represent the mean±s.e.m. of 3–4 independent determinations. *P<0.05 vs Ctrl (Student's *t*-test). Ctrl, control; PNS, prenatal stress.

We next characterized in more detail the modulation of *Ank3* in the rat model of PNS. Three genomic locations showed a different methylation status between Ctrl and PNS rats in the PFC at PND62 (Figure 2a and Supplementary Table 6). In detail, the probes located at chr20:19420344-19420403 and at chr20:19420705-19420764 were hypomethylated in PNS rats, whereas the probe located at chr20:19580638-19580697 was hypermethylated in PNS rats. qPCR analysis was used to validate the array data indicated by the arrow in Figures 2a and b, and confirmed the result obtained with MeDIP-chip (Figure 2c). We focused on this probe, as it was the only showing an increased methylation status in the stressed group, according with all the other conditions (Supplementary Tables 6 and 7).

### Postnatal developmental expression of Ankyrin-3 in the PFC of male rats

**Figure 3:**
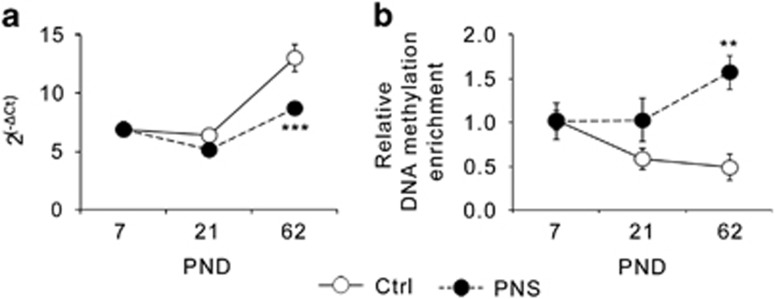
Exposure to prenatal stress in rats alters the DNA methylation status and the expression levels of Ank3 with a specific time-profile. (**a**) mRNA expression levels of Ankyrin-3 during the postnatal development in the prefrontal cortex of PNS male rats as compared with control animals (Ctrl). The data, expressed as fold change (where ΔCt is the difference between the threshold cycle of the target gene and the housekeeping gene) are the mean±s.e.m. of 6–9 independent determinations. (**b**) Relative DNA methylation enrichment of Ankyrin-3 during the postnatal development in the prefrontal cortex of PNS male rats as compared with Ctrl. The data, expressed as relative bound fraction concentration, are the mean±s.e.m. of 3–7 independent determinations. ^**^P<0.01 and ^***^P<0.001 vs Ctrl at the same postnatal age (2-way analysis of variance (ANOVA) followed by Fisher's LSD *post hoc* comparison). PND, postnatal day; PNS, prenatal stress.

Using qRT-PCR, we next examined if the altered methylation status of *Ank3* was associated with changes in its transcriptional regulation during the postnatal development until early adulthood. We found a significant effect of PNS exposure (F_1,41_=14.795, *P*<0.001), of AGE (F_2,41_=39.885, *P*<0.001), and a significant PNS × AGE interaction (F_2,41_=7.303, *P*<0.01). Indeed, as depicted in Figure 3a, Ctrl rats showed a relative stable expression of *Ank3* from PND7 to PND21, followed by a steady increase at PND62, whereas PNS rats showed statistically significant lower *Ank3* mRNA levels at PND62 (*P*<0.001), but not earlier (Figure 3a). Next, in order to establish a correlation between these changes and the epigenetic modifications at the *Ank3* gene, we investigated DNA methylation at the same gene location found to be affected using MeDIP-chip and validated with qPCR at PND62 in PNS rats. As depicted in Figure 3b, Ctrl rats showed a progressive decrease in the methylation levels of *Ank3* from PND7 to PND62, whereas PNS rats showed a stable methylation between PND7 and PND21, with a steady increase at early adulthood. Statistical analysis revealed a significant effect of PNS (F_1,29_=10.315, *P*<0.01) and a significant PNS × AGE interaction (F_2,29_=3.897, *P*<0.05), supporting the fact that PNS and Ctrl rats showed comparable methylation levels of *Ank3* at PND7 that progressively diverge, leading to a statistical difference at PND62 (*P*<0.01).

### Analysis of ANKG protein modulation following PNS exposure in the PFC of male rats at PND62

*Ankyrin-3* encodes for a protein, ANKG, which exists in different isoforms that are expressed in nearly all tissues, although with some peculiarities. It has been recently demonstrated, for example, that the 190 kDa isoform of ANKG localizes at post-synaptic level, where it interacts with both PSD95 and GLUR1.^32^ Since it has been amply demonstrated that neuropsychiatric disorders can be characterized by synaptic dysfunction,^33, 34^ we decided to investigate whether the long-term changes in *Ank3* expression observed in PNS animals may also involve alterations in the synaptic compartment.

**Figure 4:**
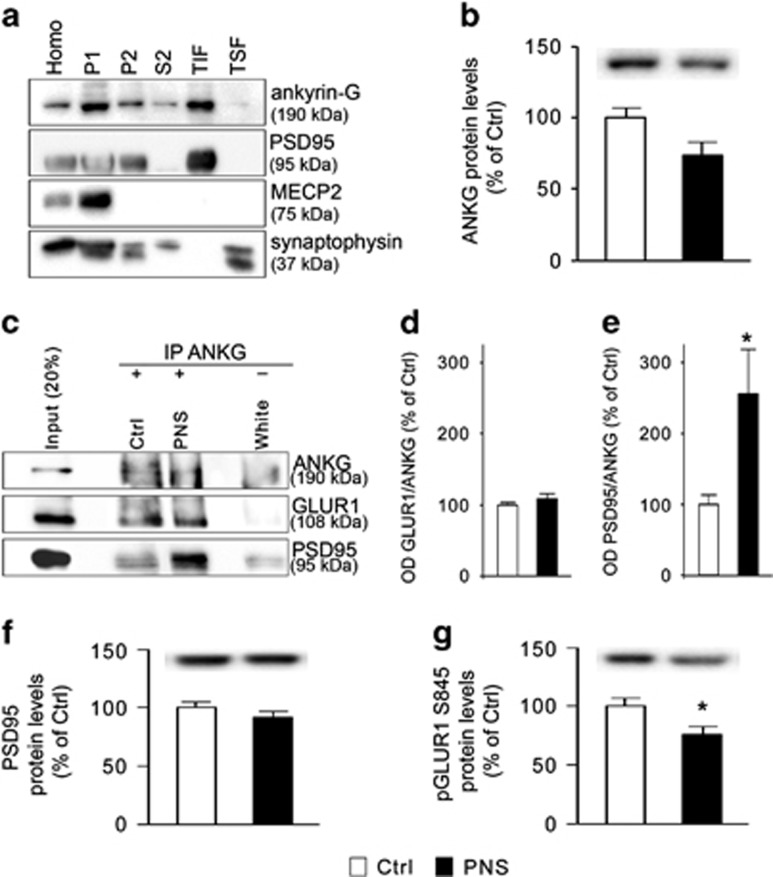
ANKG interaction with PSD95 and GLUR1: modulation following prenatal stress exposure in rats. (**a**) Western blot analysis of ANKG enrichment in different subcellular fractions, as compared with PSD95, MECP2 and synaptophysin. (**b**) Bar graph of ANKG protein levels in the crude membrane fraction from the prefrontal cortex of adult Ctrl and PNS male rats. (**c**) Western blot analysis of co-immunoprecipitation experiments of ANKG with GLUR1 or PSD95 from the prefrontal cortex of male rats at PND62. (**d** and **e**) Bar graph of GLUR1/ANKG (**d**) and PSD95/ANKG (**e**) interaction in the prefrontal cortex of Ctrl and PNS male rats at PND62. (**f**, **g**). Bar graphs of PSD95 (**f**) and pGLUR1 S845 (**g**) protein levels in the crude membrane fraction from the prefrontal cortex of adult Ctrl and PNS male rats. Data in bar graphs are presented as mean±s.e.m. of 7–8 independent determinations. *P<0.05 vs Ctrl (Student's *t*-test). ANKG, ankyrin-G; Homo, whole homogenate; P1, nuclear fraction; P2, crude membrane fraction; PNS, prenatal stress; S2, cytosolic fraction; TIF, triton insoluble fraction; TSF, triton soluble fraction.

Using western blot analysis, we first established the expression of ANKG 190 kDa in different subcellular fractions from the rat brain. As shown in Figure 4a, ANKG 190 kDa is enriched in the crude membrane fraction (P2) and in the TIF, which represents the post-synaptic compartment, as also occurred for the post-synaptic density marker PSD95, but not for MECP2, a marker of the nuclear compartment, or synaptophysin, a presynaptic marker (Figure 4a). Moreover, we found that ANKG 190 kDa levels were reduced by PNS in the P2 (−26% vs Ctrl, *P*=0.057, Figure 4b).

Seen ANKG 190 kDa enrichment in the post-synaptic compartment (Figure 4a) and its trend toward a decrease in the P2 fraction (Figure 4b), we investigated, by co-immunoprecipitation assay, whether ANKG 190 kDa interacts with key molecules localized at the post-synaptic site and if PNS exposure may alter these interactions. As shown in Figure 4c, ANKG 190 kDa interacted with PSD95 as well as with GLUR1. Interestingly, we found that while GLUR1/ANKG interaction was not affected by PNS (+9% vs Ctrl, *P*>0.05, Figure 4d), PSD95/ANKG interaction was significantly increased in the PFC of adult rats that were exposed to PNS (+155% vs Ctrl, *P*<0.05, Figure 4e), possibly leading to alterations in the functional integrity of the post-synaptic compartment. Indeed, although we did not detect changes of PSD95 protein levels in the P2 (−13% vs Ctrl, *P*>0.05, Figure 4f), we found a significant decrease of the phosphorylated (pS845) form of the obligatory AMPA GLUR1 subunit (−24% vs Ctrl, *P*<0.05, Figure 4g), a proxy for receptor activation.^53^

### Interaction between the Ank3 single-nucleotide polymorphism rs9804190 and OC on prefrontostriatal functional connectivity and behavior during working memory

Analysis of human post-mortem data, using the Braincloud data set,^54^ indicated that the *Ank3* single-nucleotide polymorphism rs9804190 was associated with *Ank3* mRNA expression in the DLPFC (F_2, 261_=6.66; *P*=0.001), with greater expression in the TT genotype as compared with CT (*P*<0.0001) and CC (*P*<0.0001).

**Figure 5:**
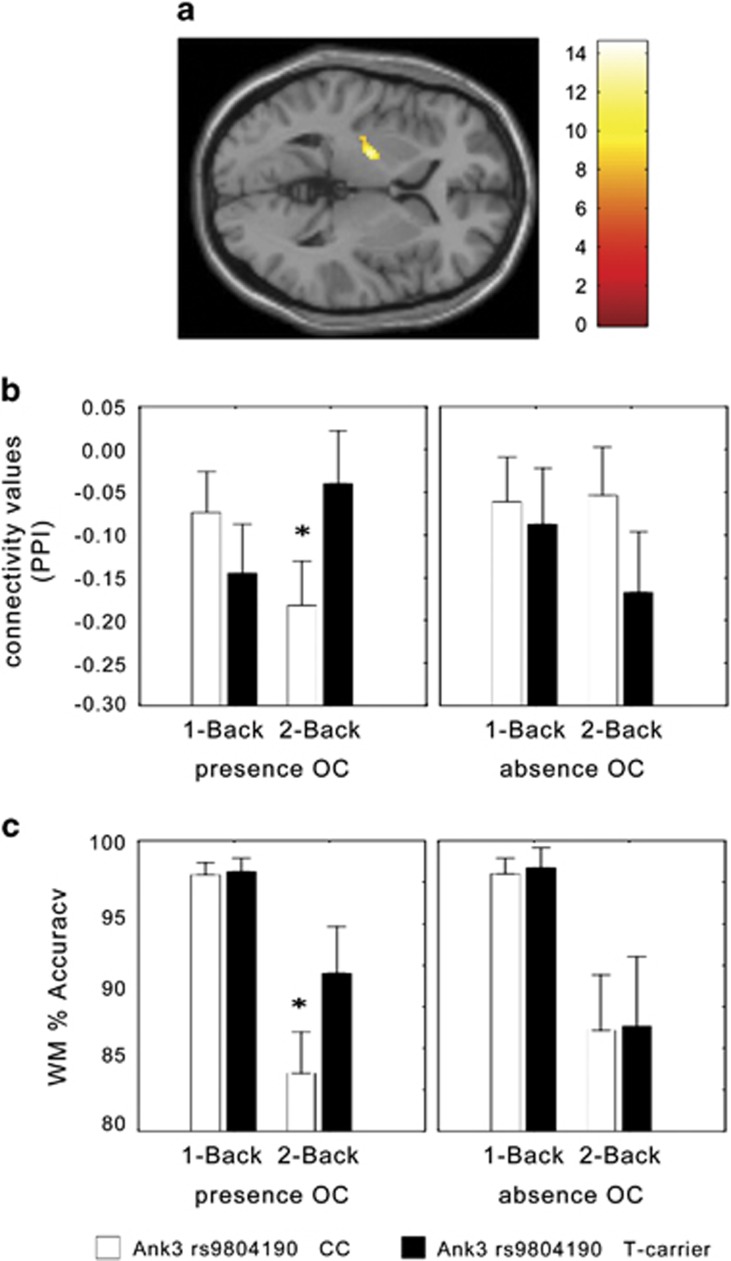
Healthy humans with a history of obstetric complications combined with functional variation in Ank3 gene, show altered prefrontostriatal connectivity and working memory performance. (**a**) Section of the brain depicting the left striatal cluster whose functional connection with the left DLPFC is associated with an Ank3 rs9804190 × OC interaction during WM processing. (**b**) Graph with functional connectivity values (arbitrary units) extracted from the cluster depicted in **a**. T carrier healthy subjects had greater connectivity strength compared with CC healthy individuals in the presence of OC. See text for statistics. (**c**) Graph showing an Ank3 rs9804190 × OC interaction on behavioral accuracy (% correct responses) during WM in healthy subjects. T carriers were associated with greater accuracy compared with subjects with the CC genotype (see text for statistics). DLPFC, dorsolateral prefrontal cortex; OC, Obstetric Complications; WM, working memory.

We next investigated the influence of rs9804190 on brain function and the possible modulation following exposure to early-life adversities, in the form of OC. fMRI results did not indicate significant main effects of OC, of rs9804190, or their interaction on brain activity. There was a rs9804190 genotype by WM load interaction in bilateral DLPFC (Right BA9, x, y, z=28, 40, 36; K=68; Z score=3.28; uncorrected *P*=0.001. Left BA9/10, x, y, z=−40, 46, 20; K=60; Z score=3.31; uncorrected *P*=0.001). Consistent with previous studies,^28, 55^ CC subjects exhibited greater recruitment of bilateral PFC during 2-back compared with T carriers. On the other hand, psychophysiological interaction analysis revealed a genotype × OC × WM load interaction on functional connectivity between left DLPFC and left striatum (*x*,*y*,*z*=−26, −16, 9; *K*=111; *Z* score=3.59; family-wise error corrected *P*=0.037). *Post hoc* analysis on values of connectivity extracted from the significant striatal cluster revealed, in presence of OC, a greater connectivity strength in T carriers as compared with CC individuals during 2-Back (*P*<0.007; Figures 5a and b). No other statistically significant difference was found in between genotype group comparisons within each load (Tukey's HSD *post hoc* test, all *P*>0.1).

Analysis of WM behavioral data indicated a main effect of genotype reaching significance (F_1.287_=3.2; *P*=0.07), with greater mean values of percent correct responses in T carriers compared with CC individuals. Furthermore, there was a trend for an interaction between genotype and load (F_1.287_=3.5; *P*=0.06], with T carrier subjects having greater mean accuracy than CC at 2-Back. No significant main effect of OC or of its interaction with WM load or rs9804190 was found (all *P*>0.1). Notably, and similarly to the psychophysiological interaction analysis of fMRI data, there was a genotype × OC × WM load interaction on percent correct responses (F_1,287_=3.88; *P*=0.049). More specifically, *post hoc* analysis demonstrated greater accuracy in T carriers compared with CC subjects at 2-Back in presence of OC (Tukey's HSD *post hoc* test *P*=0.0005). No other statistically significant difference was found between genotypic group comparisons within each load (Tukey's test, all *P*>0.9; Figure 5c). No statistically significant main effects or interaction was present on reaction time data (all *P*>0.05).

## Discussion

In the present study, using a ‘converging evolutionary' approach, we identified an association between the exposure to ELS and the methylation status of *Ank3* gene, which may support its role in the vulnerability to psychiatric disorders. *Ank3* methylation, mRNA and protein levels were indeed significantly altered in rats exposed to PNS with a specific temporal profile.^33^ Furthermore, in humans we found an interaction between a polymorphism affecting *Ank3* expression and OC, modulating prefrontostriatal functional connectivity and behavior during WM, whose deficits are manifested in several psychiatric conditions.^37^

First, in line with previous studies showing the impact of ELS on the epigenome,^49, 50, 56^ we found that PNS in rats persistently affected a large set of genes involved in specific biological functions. Subsequently, our cross-species, cross-tissues and longitudinal approach pointed to the identification of eight genes whose methylation status was affected in all the investigated cohorts, which may be relevant for the long-lasting nature of the effects brought about by ELS. Although the heterogeneity of all the conditions in this study has to be taken into account (tissues, species and timing), we strongly believe that this assortment represents the strength of our experimental procedure, seeing that we, and others, already demonstrated the high validity of these convergent approaches.^18, 22, 23, 24^

We found that some of the overlapping genes have not yet been previously associated with stress or psychopathology (*Cnga4*, *Dars2*, *Lphn2* and *Tiam1*). Instead, other groups have previously demonstrated an involvement of *Slc22a2* or *Htr4* in the efficacy of antidepressant medication,^57, 58^ whereas *Gabrg2* holds high relevance seen the involvement of GABAergic transmission in several psychiatric disorders.^51, 59^

Seen the concordance of the methylation changes across all the conditions and species investigated in the present study, we decided to focus our attention on *Ank3*, which is among the most consistently replicated and statistically significant genetic risk factor for neuropsychiatric diseases.^25, 26, 27, 28, 30^

In the adult PFC from PNS rats, we detected an increase in DNA methylation in the first intron of *Ank3*, although we also found two differentially methylated regions downstream, within intron 24, which were more methylated in Ctrl rats. Interestingly, this same location was also found to be hypermethylated in the hippocampus of PNS male rats compared with Ctrl (chr20.19580650-19580709, FDR=0.006), an effect that we validated also at mRNA level (Ctrl: 100±8 and PNS: 77±7, *P*<0.05), as in the PFC. Although hypermethylation within a promoter is frequently correlated with transcriptional silencing,^60, 61, 62^ it has been also shown that hypermethylated promoters could be activated in a transient way through chromatin remodeling, without demethylation^63^ and, on the contrary, that the promoter hypomethylation does not necessarily correlate with active transcription, as other epigenetic mechanisms could act in concert to repress gene expression.^64^ Moreover, although DNA methylation of CpG islands associated to promoter regions has been amply investigated, several studies, including our own, have shown that also intragenic and intronic regions are widely modulated after environmental stressors through methylation, thus affecting transcriptional regulation using different mechanisms, such as alternative splicing rather than alternative promoters.^62, 65^ Indeed, we showed that PNS rats have decreased total mRNA levels of *Ank3* at PND62 as a function of different methylation levels at this genomic location. Several studies, at least in mice, have shown the complexity of *Ank3* gene organization and the existence of different mRNA isoforms with a specific tissue and cellular localization and function,^66^ and based on NCBI Genbank, rats seems to share a similar transcriptional organization to mice and humans. However, in this work, using qRT-PCR, we evaluated the total mRNA levels of *Ank3*, as the differentially methylated region is within a locus included in all *Ank3* rat mRNA transcript variants. We found that *Ank3* mRNA levels are relatively stable before weaning, with a steep increase thereafter to reach adult expression, suggesting a role for *Ank3* across the postnatal neurodevelopment and in the impending maturational steps, as already hypothesized.^67, 68, 69^ Conversely, the shift between weaning and adulthood is affected in PNS rats, leading to a significant reduction of *Ank3* expression at adulthood. Interestingly, we have recently shown that the expression of *Bdnf*, a neurotrophin involved in psychiatric disorders, is significantly downregulated in PNS rats only after adolescence,^39^ thus supporting the idea that exposure to ELS may influence life-long genome adaptation thus resulting in behavioral disorders, such as bipolar disorder, depression and schizophrenia, that frequently become manifest during adolescence.^13, 70, 71, 72^

Indeed, ELS can affect the epigenome in a persistent manner.^18, 73, 74, 75^ However, it is not clear whether DNA methylation changes represent a direct consequence of the early-life experiences or a result of the psychopathological phenotype associated with perinatal adversities. We demonstrated that PNS animals not only failed to show a progressive decrease in the DNA methylation levels of *Ank3* as occurring in Ctrl, but they displayed a gradual increase after weaning. It may be inferred that two concomitant events may lead to a reduction of *Ank3* expression from weaning to adulthood: a failure of the mechanisms that reduce gene methylation (as occurring in Ctrl rats), together with an activation of *de novo* methylation as long-term consequence of stress exposure. Similar to these findings, Weaver and colleagues have previously demonstrated that maternal behavior alters the methylation status of the glucocorticoid receptor during the first week of life, thus affecting its expression in the hippocampus until adulthood.^76^

We also showed that exposure to PNS led to functional alterations in the protein encoded by *Ank3* gene, namely ANKG. We found that ANKG is enriched in the membrane compartment (P2 and TIF), as previously demonstrated using primary cortical neurons,^32^ and that PNS may affect its subcellular distribution reducing its protein levels selectively in this fraction (compartment). Furthermore, we showed that the interaction of ANKG with PSD95 was significantly increased in PNS animals, which may alter the functional integrity of the post-synaptic domain. As an example, Yaka *et al.*^77^ demonstrated that a mild form of PNS determined an increase of synaptic PSD95 levels in the hippocampus in juvenile rats, thus altering glutamate receptor clustering and leading to impairments in LTP and memory tasks. Even if we failed to detect changes of PSD95 protein levels in PNS animals, the abnormal interaction of PSD95 with ANKG 190 kDa could contribute to a disrupted AMPAR clustering and function, as also demonstrated by the reduction of the phosphorylation levels of GLUA1 S845 in PNS rats, a proxy for receptor activation.^53^ As the glutamatergic synapse represents a key player in the pathogenesis and development of neuropsychiatric disorders,^33, 34^ our data suggest that ANKG may mediate some of the detrimental effects of PNS exposure on the glutamatergic architecture.

Furthermore, we provided evidence for a functional outcome of reduced *Ank3* expression in humans. Indeed, by investigating an *Ank3* single-nucleotide polymorphism, rs9804190, which has previously been associated with heightened risk for different psychiatric disorders,^27, 28, 78, 79, 80^ we found that the C allele predicts lower post-mortem prefrontal *Ank3* expression in non-psychiatric subjects. Interestingly, CC subjects with OC had lower prefrontostriatal functional connectivity strength and lower behavioral accuracy at the higher WM demand, as compared with T carriers with OC. These results suggest that a genetic context leading to lower *Ank3* expression interacts with the presence of OC, a relevant factor of early stress, in determining sub-optimal patterns of brain functional connections and behavior during WM. Some limitations of our study need to be considered. First, the relatively small sample size in the MeDIP studies, which is due to technical issues. Second, for the animal cohorts (both rats and monkeys) only males have been used, which could limit the conclusions to a sex-specific effect. Seen the existence of sex bias in the prevalence and severity of many neurodevelopmental and psychiatric disorders, and evidence suggesting that environment has a sex-dependent effect on DNA methylation itself,^81, 82^ future studies are needed to investigate whether such alterations may characterize also the female counterpart. It is also important to mention that following ELS the methylation differences across species occur in different regions, whose functional impact on gene function remains to be established. Last, although we limited the analysis to the PFC for performing cross-species investigation, we cannot rule out the possibility that methylation changes in other key brain regions, such as the hippocampus and the basal ganglia, may mediate the long-term effect of ELS on the susceptibility to psychopathology.

The results in humans at the imaging and behavioral level, together with those obtained by our methylation analysis, suggest that *Ank3* may represent an important link between ELS and the development of psychopathology, through alterations in neuronal circuits relevant for psychiatric diseases. Moreover, given the possibility that *Ank3* expression can be modulated by pharmacological intervention ^31^ and that the behavioral phenotype induced by *Ank3* suppression in the mouse brain may be reverted by chronic lithium administration,^83^ we believe that *Ank3* may represent a novel molecular marker as well as a target for drug intervention in stress-related disorders with a neurodevelopmental origin.
