# Supplementary material for: Ankyrin-3 as a molecular marker of early-life stress and vulnerability to psychiatric disorders
Source: Transl Psychiatry. 2016 Nov 8;6(11):e943–. doi: 10.1038/tp.2016.211 (PMC5314123; doi:10.1038/tp.2016.211)
Supplement: Supplementary Methods [file tp2016211x1.docx]

**SUPPLEMENTARY DETAILED METHODS**

**Human cohort for MeDIP-chip analysis**

***Subjects***

The human cohort of mothers and their infants (n=180) has been recruited between 03/2011 and 03/2012 from two obstetric hospitals in the Rhine-Neckar Region of Germany (Mannheim, Ludwigshafen). The mothers were recruited during the third trimester of pregnancy (i.e. 4-8 weeks prior to delivery). Inclusion criteria for mothers were: Caucasian descent; main caregiver; German-speaking; and age 16 – 40 years. Exclusion criteria were: maternal hepatitis B, hepatitis C or HIV-infection; any current psychiatric disorders requiring inpatient treatment; a history, current diagnosis, of schizophrenia / psychotic disorder, or any substance dependence other than nicotine during pregnancy. Exclusion criteria for infants were: birth weight < 1 500 grams; gestational age at birth < 32 week; multiples; or any congenital diseases; malformations; deformations or chromosomal abnormality. The study protocol was approved by the Ethics Committee of the Medical Faculty Mannheim of the University of Heidelberg, and the study was conducted in accordance with the Declaration of Helsinki. All mothers provided written informed consent prior to participation.

***Assessment of exposure to stress during the third trimester of gestation and selection of samples for the genome-wide methylome analysis***

The mothers were assessed using a structured interview and a series of questionnaires in order to collect information concerning a broad range of environmental and socio-demographic risk factors, prenatal medical risk factors, general medical characteristics, and psychosocial risk factors (see supplementary Table 1a for a summary of this phenotypic assessment). Cord blood was collected immediately after birth. Eight main stressor variables derived from eight different questionnaires were selected to represent a variety of prenatal adversities, and to take three different dimensions of stress into account: a) maternal psychopathology (primarily depressive and anxiety symptoms); b) perceived stress; c) socioeconomic and psychosocial stress (for details see supplementary Table 1b). In addition, an “adversity score” was calculated by summing up the number of dichotomous stressful prenatal adverse conditions and environmental circumstances (for details see supplementary Table 1b). To obtain a homogeneous composite measure of prenatal stress, a principal component analysis (PCA) was performed. This involved the eight main stressor variables and the total adversity score as a ninth main variable. This analysis yielded a first principal component (PC1), which explained around 60% of the common variance. PC1 was then used to determine the following two extreme groups: 10 infants with extremely high levels of prenatal ELS and 10 infants with extremely low levels of prenatal ELS.

The socio-demographic and medical characteristics of the mothers and infants in the extreme groups are shown in supplementary Table 1c. The psychopathology, perceived stress, and psychosocial and socioeconomic stress status of the mothers are shown in supplementary Table 1b. For the comparison of the extreme groups, two-tailed *t*-tests for independent samples were used (SPSS® Statistics 20). The nominal level of significance was set at α = 0.05. All data and results are expressed as means ± standard deviation (SD) or as a percentage, as appropriate. The epigenome data sets of two infants in the low ELS group did not pass our quality control filters, and the group size decreased to n = 8.

**Animals and experimental paradigms**

***Monkeys and rearing procedures***

Prefrontal cortices (PFC), whole blood, CD3+ and buccal samples were obtained from rhesus monkeys (*Macaca mulatta*), randomly divided into two groups at birth resulting in different early life social and rearing experience, as previously described ^1,2^. In detail, the “mother-reared” (MR) monkeys were raised by their biological mother in a social group, whereas “surrogate peer-reared” (SPR) monkeys were reared with an inanimate surrogate as well as daily socialization periods with age-mate peers. For the first month of life, the SPR monkeys were placed in a nursery until they were able to drink milk from a bottle by themselves at which point they were transferred to a cage with their surrogate mother. At approximately 7 months of age, animals were socially housed in large, mixed sex peer groups. PFC samples were obtained from 7-year-old male monkeys ^2^. Venous blood samples were obtained from 30 days old and 2-years old monkeys at their routine medical check-up under chemical restrain (using ketamine), in order to look for stable peripheral DNA signals in response to different early environmental conditions ^3,4^. Whole blood and buccal swab samples were taken at 2 years or older age. The use of experimental animals was approved by the Institutional Animal Care and Use Committee of the NICHD. All animal experiments were carried out in accordance with the National Institute of Health Guide for the Care and Use of Laboratory Animals. All efforts were made to minimize animal suffering and to reduce the number of animals used.

***Rats and prenatal stress exposure***

Nulliparous adult female (body weight 230–260 g) and male (400 g) Sprague-Dawley rats were purchased from a commercial breeder (Charles River, Calco, Italy). Upon arrival, they were pair-housed with a same-sex conspecific with food and water available *ad libitum* (21±1 °C, 60±10% relative humidity, regular 12/12 h light/dark cycle).

After 10 days of habituation in the facility, rats were mated for 24 h and individually housed immediately thereafter. Pregnant females were randomly assigned to control (Ctrl) or prenatal stress (PNS) conditions, as previously described ^1,5,6^. In particular, PNS consisted of restraining pregnant dams in a transparent Plexiglas cylinder (7.5 cm diameter, 19 cm length) under bright light for 45 min three times daily during the last week of gestation until delivery. PNS sessions were separated by 2–3 h intervals and conducted at varying periods of the day in order to reduce habituation. Control rats were left undisturbed.

On postnatal day (PND) 1, pups from Ctrl and PNS dams were weighted, and litters were culled to 5 males and 5 females. Weaning occurred on PND21 and same sex rats were housed in groups of 3 per cage. At PND7, PND21 and PND62 one male pertaining to each litter was sacrificed.

All animal experiments were conducted according to the authorization from the Health Ministry n. 295/2012-A (20/12/2012), in full accordance with the Italian legislation on animal experimentation (D.L. 116/92) and adherent to EU recommendation (EEC Council Directive 86/609). All efforts were made to minimize animal suffering and to reduce the total number of animals used, while maintaining statistically valid group numbers.

**Molecular analyses**

***Separation of CD34+ cells from human cord blood and extraction of genomic DNA***

Human cord blood was drawn into ethylenediaminetetraacetic acid (EDTA) coated tubes immediately after birth. CD34+ cells were extracted within 24 h following delivery. Briefly, peripheral blood mononuclear cells (PBMC) were isolated by centrifuging the cord blood with Ficoll-Paque PLUS (GE Healthcare, Munich, Germany) in Leucosep tubes (Greiner Bio-One, Frickenhausen, Germany). CD34+ cells were then isolated from the PBMCs by immunomagnetic isolation using the Dynal CD34 Progenitor Cell Selection System (Life Technologies, Darmstadt, Germany) in accordance with the manufacturer´s instructions. The CD34+ cells were then stored at −80°C until DNA extraction. DNA was extracted from CD34+ cells using the Qiagen Blood Mini Kit (Qiagen, Hilden, Germany) in accordance with the instructions of the manufacturer.

***Prefrontal cortex dissection and DNA preparation from monkeys***

Animals were sedated with ketamine and brought to a deep surgical plane of anesthesia with pentobarbital administered intravenously, given to effect. A craniotomy was performed, followed by a thoracotomy and perfusion through the left ventricle of the heart for 1.5 min with a chilled, oxygenated buffer solution. The brain was then removed within 5 min and the prefrontal cortices (PFC) were flash frozen in isopentane at -55°C within 15 min of death and kept at -80°C until later analyses. The tissue consisted mostly of DLPFC and ventro-lateral PFC rostral to the caudal end of the arcuate sulcus, dorsal to the cingulate sulcus and lateral to the lateral orbital sulcus. PFC DNA was extracted using the Qiagen DNeasy kit following the protocol of the manufacturer.

***Whole blood samples and buccal cell collection from monkeys***

A small fraction (0.3 ml) of the peripheral venous blood collected for T cell isolation was used for DNA isolation with the Wizard Genomic DNA Purification kit (Promega, Madison, WI, USA) following the instructions of the manufacturer. At the same medical check-up (when the monkeys were under chemical restrain) buccal cells were collected by rubbing the inner cheeck or gum with swabs 3 times. The 3 swabs were stored in one tube at −20°C until the DNA isolation, which started with 1 hour of incubation with 60 µg proteinase K in the Nuclei Lysis Solution of the Wizard Genomic DNA Purification kit.

***Separation of CD3+ T cells from monkey peripheral blood***

For the CD3+ T cell separation, 20 ml of peripheral blood was drawn into EDTA-coated tubes from the 2-year old monkeys and stored at 4°C overnight. From 30-day old monkeys, 3 ml of blood was perfused in EDTA-coated tubes. PBMCs were isolated through centrifugation with Ficoll-Paque (GE Healthcare, Burnaby, BC, Canada). The PBMCs were than washed twice with HBSS (Life Technologies, Burlington, ON, Canada). T cells were isolated from the PBMCs by immune-magnetic isolation using CD3+ Dynabeads (Life Technologies). The beads were washed three times and incubated with the PBMCs for 45 min on a rotator at 4°C followed by a washing step (five times) with PBS/FBS. CD3+ T cells were then stored at −80°C until DNA extraction with the Wizard Genomic DNA Purification kit.

***PFC dissection and DNA/RNA preparation from rats***

After decapitation, brain tissues were dissected, immediately frozen on dry ice and stored at -80°C for later analyses. Dissections were performed according to the atlas of Paxinos and Watson ^7^. In detail, the prefrontal cortex was dissected from 2-mm thick slices (prefrontal cortex defined as Cg1, Cg3, and IL sub-regions corresponding to the plates 6–9). Isolation of DNA and RNA from the same rat specimen was performed using the AllPrep DNA/RNA Mini kit (Qiagen, Milan, Italy), in accordance with the instructions of the manufacturer.

***Methylated DNA immunoprecipitation (MeDIP) analysis***

MeDIP analyses have been performed as previously described in our studies ^1,2^. Briefly, DNA was quantified by fluorometric analysis (Qubit® 2.0 Fluorometer, Life Technologies), 2 μg of DNA were sheared by sonication and methylated DNA was immunoprecipitated using 10μg of anti-5-methyl-cytosine (Cat. No. BI-MECY-0100, Eurogentec, Fremont, CA, USA). The human study comprised n = 10 infants with high levels of ELS and n = 8 infants with low levels of ELS. The monkey study involved 4 MR and 4 SPR 7-year-old male animals for PFC analysis and 5 MR and 5 SPR 2-year-old male animals for whole blood analysis. Buccal samples were analyzed from 3 MR and 3 SPR 2-year-old male animals. For CD3+ T cells analysis, pools of DNA were used, which consisted of: (i) 10 MR or 10 SPR monkeys sampled twice, i.e. at postnatal day 30, and (ii) 6 MR or 4 SPR monkeys sampled at age 2 years. Each DNA pool was subjected to 3 parallel MeDIP analyses (that is, 3 MR and 3 SPR pools per time point). For the rat study, samples of 4 Ctrl and 4 PNS male rats sacrificed at PND62 have been used for the hybridization on the microarray, while 4-7 males per group (Ctrl or PNS) were used for the MeDIP at PND7 and PND21. The DNA antibody complex was immunoprecipitated with protein G, and the methylated DNA was re-suspended in 0.25 ml of digestion buffer (50 mM TRisHCl pH = 8; 10 mM EDTA; 0.5 % SDS) and treated with 40 μg of proteinase K overnight at 55°C. The input and bound fractions were phenol/chloroform-extracted and ethanol-precipitated. Specificity for methylated DNA and the absence of unspecific binding were validated through PCR analysis of an un-methylated (*β-actin* or *Gapdh*) and a methylated (*H19*) control gene (for primer sequences see supplementary Table 2). For MeDIP-chip, the input and bound fractions were amplified using the Whole Genome Amplification Kit (Sigma-Aldrich; St. Luis; MO; USA) and then labeled for microarray hybridization with Cy3-dUTP and Cy5-dUTP, respectively, using the CGH Enzymatic Labeling Kit (Agilent Technologies; Mississauga; ON; Canada) in accordance with the manufacturer´s instructions.

***MeDIP microarray design, hybridization, scanning and analysis***

For all microarray studies, custom promoter tiling microarray designs were used (Agilent Technologies). Probes were selected to tile all transcription start sites defined in the Ensembl database. Probes were placed approximately every 100 bp. For rat, approximately 400 K probes were selected to tile intervals covering -1 000 +200 bp around each transcription start site defined in the Ensembl database version 62 (Agilent design: 030107). Any genomic coordinates are given with respect to the rn4 (RGSC 3.4) rat genome assembly. For monkey PFC, the microarray design has already been described ^2^. Approximately 240 K probes were selected to tile all transcription start sites defined in the Ensembl database version 46 (Agilent design: 018390). Any genomic coordinates are given with respect to the rheMac2 monkey genome assembly. For monkey whole blood, CD3+ and buccal cells, approximately 400K probes were selected to tile - 1 800 + 400 bp around each transcription start site defined in the Ensembl database version 64 (Agilent design: 0375811). Genomic coordinates are also given with respect to the rheMac2 genome assembly. For human CD34+ cells, the microarray design has already been described ^1^. Approximately 400K probes were selected to tile – 2000 + 400 bp around each transcription start site defined in the Ensembl database version 59 (Agilent design: 024757). Genomic coordinates are given with respect to the hg19 (GRCh37) human genome assembly.

Probe intensities were extracted from scanned microarray images using Agilent's Feature Extraction 10.7.3.1 Image Analysis Software and analyzed using the R software environment for statistical computing (R Development Core Team, 2007) (http://www.r-project.org/). Log-ratios of the bound (Cy5) and input (Cy3) microarray channel intensities were computed for each microarray.  Microarray quality was assessed using plots generated by the feature extraction software in addition to correlation matrix visualizations. Microarrays were normalized using quantile-normalization ^8^. Estimates of DNA methylation levels based on microarray probe intensities were obtained using a Bayesian deconvolution algorithm ^9^. Differential methylation between groups was determined in two stages. The first stage used linear models implemented in the ‘limma’ package ^10^ of Bioconductor ^11^ to compute a modified t-statistic at the individual probe level. As in typical microarray studies, the number of sample profiles is small so there is little information per probe from which to estimate probe variance. Hence, the modified t-statistic for each probe makes use of variance estimates derived from the other probes on the microarray. Correlation between technical replicates was modeled as a random effect using the “block” variable. An individual probe was called differentially methylated if the significance of its t-statistic was at most 0.05 (uncorrected for multiple testing) and the associated difference of log-normalized means between the groups was at least 0.5. In the second stage, differential statistics per promoter were derived from the t-statistics of the probes within the promoter. In particular, the Wilcoxon rank-sum test was used to identify enrichment for probes within the promoter having large positive or large negative t-statistics. The resulting promoter p-values were adjusted for multiple testing by calculating false discovery rates using the Benjamini-Hochberg algorithm. A promoter was called differentially methylated if its false discovery rate was at most 0.2 and it contained at least one probe called differentially methylated, as defined above.

***Validation using qPCR***

Gene-specific quantitative real-time PCR validation of microarray (Q-MeDIP) was performed on the amplified-bound fraction for the same subjects used for microarray experiments. QPCR was performed in 1X Power SYBR Green Master Mix (Life technologies) using 4 - 10ng of DNA and 1.5 - 4 μM gene specific primers (supplementary Table 2) which were designed using Primer 3 (http://primer3.ut.ee/) free software. Relative enrichment of triplicate reactions was determined after normalizing from the input fraction in each sample using the 2^(-ΔΔCt)^ method. All data are expressed as group means ± SEM. To test for statistical significance the Student’s *t* test was used (two-tailed), and the alpha level was set at 0.05.

***QRT-PCR and analyses of mRNA levels in rats***

Total RNA was quantified by spectrophotometric analysis (NanoDrop, Thermo Scientific) and samples were processed for real-time polymerase chain reaction (qPCR) to assess mRNA levels. An aliquot of each sample was treated with DNase to avoid DNA contamination.

RNA was analyzed by Taqman qRT-PCR instrument (CFX384 real time system, Bio-Rad Laboratories) using the iScriptTM one-step RT-PCR kit for probes (Bio-Rad Laboratories). Samples were run in 384 well formats in triplicate as multiplexed reactions with a normalizing internal control (*36b4*). We choose *36b4* as internal standard for gene expression analyses since its expression was not affected by developmental changes and it was not altered by prenatal stress.

Probe and primer sequences of *36b4* (Forward Primer: TCAGTGCCTCACTCCATCAT; Reverse Primer: AGGAAGGCCTTGACCTTTTC; Taqman Probe: TGGATACAAAAGGGTCCTGG) and *Ankyrin-3* (Forward Primer: TCCTACAGAACGACACCAATG; Reverse Primer: CTCGGTTTAACAGCAACGTG; Taqman Probe: TGGCTTCACCCCACTCCACATAG) were purchased from Eurofins Genomics (Vimodrone, Italy).

Thermal cycling was initiated with an incubation at 50°C for 10 min (RNA retrotranscription) and then at 95°C for 5 min (TaqMan polymerase activation). After this initial step, 39 cycles of PCR were performed. Each PCR cycle consisted of heating the samples at 95°C for 10 s to enable the melting process and then for 30 s at 60°C for the annealing and extension reaction. Relative target gene expression was calculated according to the 2^(-ΔΔCt)^ method. Data are expressed as group means ± SEM. To test for statistical significance, a two-way ANalysis Of VAriance (ANOVA), followed by Fisher’s LSD post-hoc comparisons was performed using SPSS for Mac OS X (Release 22.0.0.0), with ‘PNS exposure’ or ‘AGE’ as independent factors. A probability level of *p*<0.05 was taken as significant in every test.

**Biochemical analyses in rats**

***Protein extraction, SDS-PAGE and western blot analysis***

The prefrontal cortices of the second rat hemisphere were homogenized in a Teflon-glass potter in ice-cold 0.32 M sucrose buffer containing 1 mM HEPES, 1 mM MgCl_2_, 1 mM NaHCO_3_, and 0.1 mM phenylmethylsulfonyl fluoride, pH=7.4, in the presence of a complete set of protease (Roche) and phosphatase (Sigma-Aldrich) inhibitors. The homogenized tissue was centrifuged at 1 000g for 10 min to separate a pellet (P1) enriched in nuclear components from the supernatant (S1). S1 was then centrifuged at 13 000g for 15 min to obtain a clarified fraction of cytosolic proteins (S2). The pellet (P2), corresponding to the crude membrane fraction, was resuspended in 20 mM HEPES in the presence of a complete set of protease (Roche) and phosphatase (Sigma-Aldrich) inhibitors. An aliquot of the P2 was centrifuged at 100 000 g for 1 h. The pellet (P3) was resuspended in buffer containing 150 mM KCl, 0,5% Triton X-100 and 1mM HEPES in the presence of a complete set of protease (Roche) and phosphatase (Sigma-Aldrich) inhibitors with a Teflon-polycarbonate potter and centrifuged at 100 000 g for 1 h. The resulting supernatant (S4), referred as Triton X-100-soluble fraction (TSF), was stored at -20°C, while the pellet (P4), referred as the Triton X-100 insoluble fraction (TIF) which is enriched in the postsynaptic compartment, was homogenized in a glass-glass potter in 20 mM HEPES in the presence of a complete set of protease (Roche) and phosphatase (Sigma-Aldrich) inhibitors and stored at -20°C in the presence of glycerol 30%.

Total protein content was measured according to the Bradford Protein Assay procedure (Bio-Rad Laboratories), using bovine serum albumin as a calibration standard.

Qualitative enrichment analysis of ankyrin-G 190 kDa was performed on the above-mentioned subcellular fractions, while semi-quantitative protein analyses were performed in the crude membrane fraction (P2). Equal amounts of total protein (5 μg for the TIF and 20 μg for all the other subcellular compartments) were run under reducing conditions (150 V at room temperature) on an SDS-polyacrylamide gel (7-8 % SDS-PAGE) and then electrophoretically transferred onto nitrocellulose membranes at 250 mA for 2 h at 4°C. Transfer efficiency was controlled by Ponceau S staining and pre-stained protein standards. Unspecific binding sites were blocked for 1 h in 10% non-fat dry milk in Tris-buffered saline solution containing 0.1% Tween-20 (TBS-T). Membranes were then incubated at 4°C over night with the following primary antibodies: rabbit polyclonal anti-ANKG primary antibody (Cat. No. sc-28561, Santa Cruz Biotechnology, 1:500 in 5 % non-fat dry milk in TBS-T), rabbit monoclonal anti-PSD95 primary antibody (Cat. No. 3450, Cell Signaling, 1:4 000 in 3 % non-fat dry milk in TBS-T), rabbit monoclonal anti-MECP2 primary antibody (Cat. No. 3456P, Cell Signaling, 1:1 000 in 5 % bovine serum albumin in TBS-T), rabbit monoclonal anti-synaptophysin primary antibody (Cat. No. D35E7, Cell Signaling, 1:1 000 in 3 % non-fat milk in TBS-T) and rabbit polyclonal anti-GLUR1 phosphoSer845 primary antibody (Cat. No. AB5849, Millipore, 1:500 in 5 % bovine serum albumin in TBS-T).

Membranes were then washed with TBS-T and incubated for 1 h at room temperature with a peroxidase-conjugated anti-rabbit IgG (Cat. No. BK4074S, Cell-Signaling Technology) (ANKG, 1:4 000 in 5 % non-fat milk in TBS-T; PSD95, 1:8 000 in 3 % non-fat milk in TBS-T; MECP2, 1:5 000 in 5 % non-fat milk in TBS-T; synaptophysin, 1:10 000 in 3 % non-fat milk in TBS-T; pGLUR1 S845, 1:2 000 in 3 % non-fat milk in TBS-T). Immunocomplexes were visualized by chemiluminescence using the ECL Western Blotting kit (Euroclone) according to the manufacturer’s instructions using the Chemidoc MP Imaging System (Bio-Rad Laboratories) and protein levels were calculated by measuring the optical density of the autoradiographic bands using Image-Lab software (Bio-Rad Laboratories). Results were standardized using β-Actin as control protein that was detected by evaluating the band density at 43 kDa after blocking the membranes with 10% non-fat dry milk and probing it with monoclonal mouse primary antibody anti-β-Actin (Cat. No. A5441, Sigma-Aldrich, 1:10 000 in 3 % non-fat dry milk in TBS-T), followed by the incubation with a peroxidase-conjugated antibody anti-mouse IgG (Cat. No. A4416, Sigma-Aldrich, 1:10 000 in 3 % non-fat dry milk in TBS-T dilution). To ensure that autoradiographic bands were in the linear range of intensity, different exposure times were used. Actin was employed as an internal standard because its expression is not regulated by the experimental paradigm used.

Data are expressed as group means ± SEM. To test for statistical significance the Student’s *t* test was used (two-tailed), and the alpha level was set at 0.05.

***Coimmunoprecipitation assay***

Co-immunoprecipitation assays were performed as in Vastagh et al. ^12^ with the introduction of some methodological modifications. In detail, Aliquots of total cell homogenate (200 μg) were incubated in RIA buffer (400 mM NaCl, 20 mM EDTA, 20 mM Na_2_HPO_4_, 1% Nonidet P-40) and 0.1% sodium dodecylsulfate (SDS) with 3 μg of antibody against ankyrin-G (Cat. No. 75-146, Neuromab, clone N106/36) overnight at 4°C on a wheel, except for the ‘white’, in which the antibody was not added. Protein A/G agarose beads (Santa Cruz Biotechnology) were added and incubated for 2 h at 25°C on the wheel. Beads were collected by gravity and washed in RIA buffer containing 0.1 % SDS three times. Laemmli sample buffer was then added to the samples and to the input (sample that did not undergo the IP and that were prepared with 10% of the starting material used for coimmunoprecipitation experiments, that is 20 μg of homogenate). The mixture was boiled for 10 minutes at 100°C. Beads were collected by gravity and the supernatant of the immunoprecipitated samples, of the input and of the white was loaded onto a 7% acrylamide/bisacrylamide gel for SDS–PAGE. The proteins were run at 30 mA and then electrophoretically transferred onto nitrocellulose membranes at 250 mA for 2 h at 4°C. We next performed Western blot analysis using mouse monoclonal anti-GLUR1 primary antibody (Cat. No. MAB-2263, Millipore, 1:1 000 in 3 % non-fat milk in TBS-T) and rabbit monoclonal anti-PSD95 primary antibody (Cell Signaling, 1:4 000 in 3 % non-fat dry milk in TBS-T). After, PSD95 was incubated for 1 h at room temperature with a peroxidase-conjugated anti-rabbit IgG (Sigma-Aldrich, 1:8 000 in 3 % non-fat milk in TBS-T), while GLUR1 was incubated with a peroxidase-conjugated antibody anti-mouse IgG (Sigma-Aldrich, 1:3 000 in 3 % non-fat dry milk in TBS-T). Results were standardized using ANKG as normalizing protein that was detected by evaluating the band density at 190 kDa after blocking the membranes with 10 % non-fat dry milk and probing it with rabbit polyclonal anti-ankyrin-G primary antibody (Santa Cruz Biotechnologies, 1:400 in 3 % non-fat milk in TBS-T), followed by the incubation with a peroxidase-conjugated antibody anti-rabbit IgG (Sigma-Aldrich, 1:3 000 in 5 % non-fat dry milk in TBS-T dilution). To ensure that autoradiographic bands were in the linear range of intensity, different exposure times were used.

Data are expressed as group means ± SEM. To test for statistical significance, the Student’s *t* test was used (two-tailed), and the alpha level was set at 0.05.

**Bioinformatics analysis**

***Venn diagrams***

Venn diagrams (http://bioinformatics.psb.ugent.be/webtools/Venn/) were used to show all the possible overlaps between the datasets obtained from the different MeDIP-chip analyses performed in our different paradigms of adversities early in life.

***Ingenuity Pathway Analysis***

Ingenuity Pathway Analysis (IPA) bioinformatics resource (www. ingenuity.com) was used to analyze, integrate and interpret the lists of genes obtained from -omics experiments. IPA scores our dataset of interest against pathways in the Ingenuity Knowledge Base which houses a large database of biological and chemical relationships extracted from scientific literature, integrating data from a variety of experimental platforms. For each analysis, IPA ranks each function or pathway using the right tailed Fisher Exact Test, which measures the likelihood that the lists of genes are associated with a certain pathway/function.

**Interaction between functional variation in *Ank3* and obstetric complications on working memory processing**

***Post-mortem investigation of Ank3 functional variation***

Earlier results have indicated association of an intronic SNP in the *Ank3* gene, i.e. rs9804190, with gene expression levels in the superior temporal gyrus of patients with schizophrenia ^13^. We therefore used the Braincloud dataset available at http://braincloud.jhmi.edu ^14^ to probe the association between rs9804190 and *Ank3* transcription levels in human *post mortem* prefrontal cortex (Brodmann Area 46). In particular, we investigated 268 brains of Caucasian (N=112), African American (N=146), Asiatic (N=4) and Hispanic (N=6) non-psychiatric individuals (supplementary Table 3). Details of tissue acquisition, handling, processing, dissection, clinical characterization, neuropathological examinations, RNA extraction and quality control measures were described previously ^14,15^.

RNA from prefrontal grey matter was analyzed using spotted oligonucleotide microarrays yielding data from 30 176 gene expression probes and allowing us to focus on *Ank3* mRNA expression. In particular, total RNA was extracted, amplified and fluorescently labeled. Reference RNA was pooled from all samples and treated identically to sample RNAs. After normalization ^16^, log2 intensity ratios were further adjusted to reduce the impact of known and unknown sources of systematic noise on gene expression measures using surrogate variable analysis ^14,17^.

Data relative to 23 different transcription probes binding *Ank3* gene were available in the Braincloud dataset. We selected the hHA034365 probe based on its specific affinity for *Ank3* brain-specific isoform. In addition, differently from other available probes, the one on which we focused our analysis recognizes a central transcript region of the gene, thus softening possible artifacts due to low RNA integrity.

DNA from cerebellar tissue was investigated with Illumina Bead Chips producing 625,439 SNP genotypes called using the Bead Express software for each subject as previously described ^14,17^. We focused our attention on rs9804190 genotype for association analysis. Thus, ANCOVA was performed, with rs9804190 genotype as the independent variable, *ANK3* mRNA expression as the dependent variable, and non-matched variables between groups (i.e. age, sex, RNA integrity number) and ethnicity as covariates of no interest. A statistical threshold of *p*<0.05 was used for this analysis.

***In vivo fMRI and behavioral studies***

***Subjects***

306 healthy Caucasian subjects from the region of Apulia, Italy, were enrolled in a behavioral study and were evaluated with the Structured Clinical Interview for DSM-IV ^18^ to exclude any psychiatric disorder. Further exclusion criteria were: history of drug or alcohol abuse, active drug use in the past year, head trauma with loss of consciousness, and any significant medical condition revealed by clinical assessment and magnetic resonance imaging. Handedness (Edinburgh Inventory) ^19^ and total IQ (WAIS-R) were also measured. All subjects participating to the protocol signed an informed consent before entering the study. The protocol was approved by the local ethics committee of the “Azienda Ospedaliero-Universitaria Consorziale Policlinico di Bari”. 174 of these individuals (supplementary Table 4) also participated to a fMRI study. In both studies, all individuals performed the 1- and 2-back versions of the N-back task, eliciting two loads of working memory (WM) processing and were genotyped for rs9804190 (see below). Furthermore, mothers of all individuals completed the McNeil-Sjöström Scale ^20^, which allowed to split individuals based on the Obstetric Complications (OC) score (see below) ^21^. Because of the small number of subjects carrying the TT genotype, these subjects and those carrying the GT genotype were collapsed in a single group (T carriers) in both the fMRI and the behavioral studies. Sample sizes were: a) in the fMRI sample, 57 CC with OC, 48 CC without OC, 38 T carriers with OC, 31 T carriers without OC; b) in the behavioral study sample, 115 CC with OC, 66 CC without OC, 86 T carriers with OC, 39 T carriers without OC. All groups were matched for socio-demographic variables (i.e. age, gender, total IQ, handedness, Hollingshead index) (*p* > 0.05).

***N-Back task***

Briefly, ‘N-back’ refers to how far back in the sequence of stimuli the subject had to recall. The stimuli consisted of numbers (1–4) shown in random sequence and displayed at the points of a diamond-shaped box. There was a visually paced motor task, which also served as a non-memory guided control condition (0-Back) that simply required subjects to identify the stimulus currently seen. In the working memory (WM) conditions, the task required recollection of a stimulus seen one (1-Back) or two stimuli (2-Back) previously while continuing to encode additionally incoming stimuli. Performance data were recorded as the percentage of correct responses (accuracy) and reaction time (ms).

During the fMRI data acquisition, subjects performed a blocked design version of the N-back task. In particular, there was a non memory-guided control condition (0-Back) that simply required subjects to identify the stimulus currently seen and the 1-Back and 2-Back conditions. The stimuli were arranged in eight blocks, each lasting 30 seconds: four blocks of the control condition alternating with four blocks of each WM condition. Stimuli were presented via a back-projection system. Responses were recorded through a fiber optic response box allowing measurement of percent accuracy and reaction time (in milliseconds). All subjects were trained on the task prior to the fMRI session.

***Ank3 rs9804190 genotype determination***

Genomic DNA of all individuals was isolated from whole blood using the QIAamp DNA Blood Maxi Kit (Qiagen, [Venlo](http://en.wikipedia.org/wiki/Venlo), [Netherlands](http://en.wikipedia.org/wiki/Netherlands)). Rs9804190 genotype determination was performed using 20 ng gDNA, which was amplified according to manufacturer's directions through a TaqMan® SNP Genotyping Probe (Assay ID C_29667085_10) (Life Technologies, [Carlsbad](https://www.google.it/search?rlz=1C1ASRM_enIT612IT613&es_sm=122&biw=1920&bih=955&q=carlsbad&stick=H4sIAAAAAAAAAGOovnz8BQMDgwsHnxCXfq6-gUlVRUp8rhIHiF1kUp6npZWdbKWfX5SemJdZlViSmZ-HwrHKSE1MKSxNLCpJLSrWmve6Kdz946I1T64kb_72S-YVv3QKAHDwmgFhAAAA&sa=X&ei=aY_sVPv4JcTYap_dgdAD&sqi=2&ved=0CJQBEJsTKAEwEA), [CA](https://www.google.it/search?rlz=1C1ASRM_enIT612IT613&es_sm=122&biw=1920&bih=955&q=california&stick=H4sIAAAAAAAAAGOovnz8BQMDgwsHnxCXfq6-gUlVRUp8rhIHiG2YZ16opZWdbKWfX5SemJdZlViSmZ-HwrHKSE1MKSxNLCpJLSo-9GuRaiSL_N-PXgdL-Hla7v5v3dwJAFQmsKhhAAAA&sa=X&ei=aY_sVPv4JcTYap_dgdAD&sqi=2&ved=0CJUBEJsTKAIwEA), [USA)](https://www.google.it/search?rlz=1C1ASRM_enIT612IT613&es_sm=122&biw=1920&bih=955&q=usa&stick=H4sIAAAAAAAAAGOovnz8BQMDgysHnxCXfq6-gUlVRUp8rhIniG2ZbF5uoKWVnWyln1-UnpiXWZVYkpmfh8KxykhNTCksTSwqSS0q_v72Qrb3JLsr5f6M79sjfv6-eHgZAwBeCup1YgAAAA&sa=X&ei=aY_sVPv4JcTYap_dgdAD&sqi=2&ved=0CJYBEJsTKAMwEA) in a MJ Mini Opticon thermal cycler (Bio-Rad, [Hercules, CA, USA)](https://www.google.it/search?rlz=1C1ASRM_enIT612IT613&q=california+hercules&stick=H4sIAAAAAAAAAOPgE-LSz9U3MC4wzDVPUeIAsQsrCwu1tLKTrfTzi9IT8zKrEksy8_NQOFYZqYkphaWJRSWpRcUALCJywkQAAAA&sa=X&ved=0ahUKEwj4tZCqu7rJAhXEiQ8KHXluBwoQmxMIiQEoATAQ). Genotype calls were assessed with CFX Manager™ Software (Bio-Rad, [Hercules, CA, USA)](https://www.google.it/search?rlz=1C1ASRM_enIT612IT613&q=california+hercules&stick=H4sIAAAAAAAAAOPgE-LSz9U3MC4wzDVPUeIAsQsrCwu1tLKTrfTzi9IT8zKrEksy8_NQOFYZqYkphaWJRSWpRcUALCJywkQAAAA&sa=X&ved=0ahUKEwj4tZCqu7rJAhXEiQ8KHXluBwoQmxMIiQEoATAQ). Minor allele homozygotes and heterozygous subjects were collapsed in one group in order to obtain sufficient sample sizes for the fMRI and behavioral analyses.

***Obstetric complications (OC) collection and scoring***

OC scores were determined through the McNeil-Sjöström Scale ^20^ that was administered to mothers of all participants. The purpose of the scale is to provide a standardized, functional system for the weighting of OC. Several hundred specific OC are included together with severity weights (representing an ordinal scale). The McNeil-Sjöström scale categorizes hundreds of different complications, rating each of them on a scale of 6 points, depending on its potential to determine a damage on the central nervous system of the newborn: 1) not harmful or relevant; 2) not likely harmful or relevant; 3) potentially but not clearly harmful or relevant; 4) potentially and clearly harmful or relevant; 5) potentially, clearly and greatly harmful/relevant; 6) very great harm to or deviation in offspring. Consistent with earlier studies ^22^ presence of OC were categorized depending on presence of at least one serious OC (severity score ≥3).

***fMRI data acquisition***

Blood oxygen level-dependent (BOLD) fMRI data were collected with a GE Signa 3T scanner (GE Healthcare), equipped with a standard quadrature head coil. A gradient-echo planar imaging sequence (repetition time, 2000 ms; echo time, 30 ms; thickness 4 mm; gap, 1 mm; flip angle, 90°; field of view, 24 cm; matrix, 64 x 64; 120 volumes for each run, 20 interleaved axial slices; duration: 4 min and 8 s) was used. The first four scans were discarded to allow for T1 equilibration effect.

***fMRI data processing***

Analysis of the fMRI data was completed using Statistical Parametric Mapping 8 (SPM8; <http://www.fil.ion.ucl.ac.uk/spm>). Images of each subject were slice timing corrected and pre-processed. In particular, standard procedures of realignment to the mean image were performed using the Realign and Unwarp algorithm provided in SPM8 in order to compensate for non-linear signal distortions potentially induced by head motion. Furthermore, movement parameters were extracted to eventually exclude subjects with excessive head motion (>2 mm translation, >2° rotation). Realigned images were resliced to a 2 mm isotropic voxel size, spatially normalized into a standard space (Montreal Neurological Institute template) with a 12 parameter affine model and smoothed using a 6 mm full-width half-maximum isotropic gaussian kernel to minimize noise and to account for residual inter-subject differences. A box car model convolved with the hemodynamic response function at each voxel was modeled. Linear contrasts were then computed producing a t statistical map for the 1 and 2 Back conditions, assuming the 0-Back condition as baseline.

***Psychophysiological interaction (PPI) analysis***

We next explored the interaction between *ANK3* and OC on prefronto-striatal connectivity. With this aim, psychophysiological interaction (PPI) analysis ^23^ was performed for each subject. In particular, we used a 5 mm ROI centered on the peak activity (x=-48, y=38, z=30) in left DLPFC as seed region. PPI was calculated using the first eigenvariate of individual raw activation time courses, extracted by using a singular value decomposition method from a volume of interest centered on the subject-specific peak cluster within the seed region. These time courses were then mean centered, high-pass filtered and deconvolved. A general linear model was computed using three regressors: a physiological regressor (the time course response in the volume of interest), a psychological regressor (task design) and a PPI term, calculated as the cross-product of the previous two terms. Second-level random effects multiple regression were performed to investigate *Ank3* x OC interaction on prefrontal activity during performance of the 1- and 2-back WM tasks, using task load (1- and 2-back) as the repeated measures factor, and *Ank3* rs9804190 genotype as well as OC (absence/presence) as the between-subjects factor. We used a statistical threshold of *p*<0.05, family-wise error (FWE) small volume corrected for the left DLPFC, whose role is crucial in WM processing ^24^ and the greater involvement of the left portion of DLPFC during WM tasks eliciting both verbal and visuo-spatial processing ^25,26^.

***Analysis of behavioral data***

A repeated measure factorial ANOVA was performed, with genotype and OC presence/absence as the between-subjects factors, load (1- and 2-back) as the repeated-measures factor and behavioral accuracy (% of correct responses) or reaction time as the dependent variable. Tukey’s test was used for post-hoc analyses.

***References***

1. Nieratschker V, Massart R, Gilles M, Luoni A, Suderman MJ, Krumm B *et al.* MORC1 exhibits cross-species differential methylation in association with early life stress as well as genome-wide association with MDD. *Transl Psychiatry* 2014; **4:e429.**

2. Provencal N, Suderman MJ, Guillemin C, Massart R, Ruggiero A, Wang D *et al.* The signature of maternal rearing in the methylome in rhesus macaque prefrontal cortex and T cells. *J Neurosci* 2012; **32**(44)**:** 15626-15642.

3. Massart R, Suderman M, Provencal N, Yi C, Bennett AJ, Suomi S *et al.* Hydroxymethylation and DNA methylation profiles in the prefrontal cortex of the non-human primate rhesus macaque and the impact of maternal deprivation on hydroxymethylation. *Neuroscience* 2014; **268:139-48.**

4. Nemoda Z, Massart R, Suderman M, Hallett M, Li T, Coote M *et al.* Maternal depression is associated with DNA methylation changes in cord blood T lymphocytes and adult hippocampi. *Transl Psychiatry* 2015; **5:e545.**

5. Luoni A, Berry A, Calabrese F, Capoccia S, Bellisario V, Gass P *et al.* Delayed BDNF alterations in the prefrontal cortex of rats exposed to prenatal stress: preventive effect of lurasidone treatment during adolescence. *Eur Neuropsychopharmacol* 2014; **24**(6)**:** 986-995.

6. Maccari S, Piazza PV, Kabbaj M, Barbazanges A, Simon H, Le Moal M. Adoption reverses the long-term impairment in glucocorticoid feedback induced by prenatal stress. *J Neurosci* 1995; **15**(1 Pt 1)**:** 110-116.

7. Paxinos G, Watson C. *The rat brain in stereotaxis coordinates*. Academic Press: New York1996.

8. Bolstad BM, Irizarry RA, Astrand M, Speed TP. A comparison of normalization methods for high density oligonucleotide array data based on variance and bias. *Bioinformatics* 2003; **19**(2)**:** 185-193.

9. Down TA, Rakyan VK, Turner DJ, Flicek P, Li H, Kulesha E *et al.* A Bayesian deconvolution strategy for immunoprecipitation-based DNA methylome analysis. *Nat Biotechnol* 2008; **26**(7)**:** 779-785.

10. Smyth GK, Michaud J, Scott HS. Use of within-array replicate spots for assessing differential expression in microarray experiments. *Bioinformatics* 2005; **21**(9)**:** 2067-2075.

11. Gentleman RC, Carey VJ, Bates DM, Bolstad B, Dettling M, Dudoit S *et al.* Bioconductor: open software development for computational biology and bioinformatics. *Genome Biol* 2004; **5**(10)**:** R80.

12. Vastagh C, Gardoni F, Bagetta V, Stanic J, Zianni E, Giampa C *et al.* N-methyl-D-aspartate (NMDA) receptor composition modulates dendritic spine morphology in striatal medium spiny neurons. *J Biol Chem* 2012; **287**(22)**:** 18103-18114.

13. Roussos P, Katsel P, Davis KL, Bitsios P, Giakoumaki SG, Jogia J *et al.* Molecular and genetic evidence for abnormalities in the nodes of Ranvier in schizophrenia. *Arch Gen Psychiatry* 2012; **69**(1)**:** 7-15.

14. Colantuoni C, Lipska BK, Ye T, Hyde TM, Tao R, Leek JT *et al.* Temporal dynamics and genetic control of transcription in the human prefrontal cortex. *Nature* 2011; **478**(7370)**:** 519-523.

15. Lipska BK, Deep-Soboslay A, Weickert CS, Hyde TM, Martin CE, Herman MM *et al.* Critical factors in gene expression in postmortem human brain: Focus on studies in schizophrenia. *Biological psychiatry* 2006; **60**(6)**:** 650-658.

16. Colantuoni C, Henry G, Zeger S, Pevsner J. SNOMAD (Standardization and NOrmalization of MicroArray Data): web-accessible gene expression data analysis. *Bioinformatics* 2002; **18**(11)**:** 1540-1541.

17. Leek JT, Storey JD. Capturing heterogeneity in gene expression studies by surrogate variable analysis. *PLoS genetics* 2007; **3**(9)**:** 1724-1735.

18. Williams JB, Gibbon M, First MB, Spitzer RL, Davies M, Borus J *et al.* The Structured Clinical Interview for DSM-III-R (SCID). II. Multisite test-retest reliability. *Arch Gen Psychiatry* 1992; **49**(8)**:** 630-636.

19. Oldfield RC. The assessment and analysis of handedness: the Edinburgh inventory. *Neuropsychologia* 1971; **9**(1)**:** 97-113.

20. McNeil TF, Cantor-Graae E, Sjostrom K. Obstetric complications as antecedents of schizophrenia: empirical effects of using different obstetric complication scales. *J Psychiatr Res* 1994; **28**(6)**:** 519-530.

21. Verdoux H, Sutter AL. Perinatal risk factors for schizophrenia: diagnostic specificity and relationships with maternal psychopathology. *Am J Med Genet* 2002; **114**(8)**:** 898-905.

22. Verdoux H, Sutter AL, Glatigny-Dallay E, Minisini A. Obstetrical complications and the development of postpartum depressive symptoms: a prospective survey of the MATQUID cohort. *Acta Psychiatr Scand* 2002; **106**(3)**:** 212-219.

23. Friston KJ, Buechel C, Fink GR, Morris J, Rolls E, Dolan RJ. Psychophysiological and modulatory interactions in neuroimaging. *Neuroimage* 1997; **6**(3)**:** 218-229.

24. Callicott JH, Mattay VS, Bertolino A, Finn K, Coppola R, Frank JA *et al.* Physiological characteristics of capacity constraints in working memory as revealed by functional MRI. *Cereb Cortex* 1999; **9**(1)**:** 20-26.

25. Rottschy C, Langner R, Dogan I, Reetz K, Laird AR, Schulz JB *et al.* Modelling neural correlates of working memory: a coordinate-based meta-analysis. *Neuroimage* 2012; **60**(1)**:** 830-846.

26. Wager TD, Smith EE. Neuroimaging studies of working memory: a meta-analysis. *Cogn Affect Behav Neurosci* 2003; **3**(4)**:** 255-274.
