# Supplementary material for: Ankyrin-3 as a molecular marker of early-life stress and vulnerability to psychiatric disorders
Source: Transl Psychiatry. 2016 Nov 8;6(11):e943–. doi: 10.1038/tp.2016.211 (PMC5314123; doi:10.1038/tp.2016.211)
Supplement: Supplementary Tables [file tp2016211x2.pdf]

Supplementary Table 1

Phenotypic characteristics of mothers and infants

Supplementary Table 1a: Summary of the phenotypic assessment of mothers and infants at two time points early in life

	**Prenatal / 3rd Trimester**	**Perinatal / birth**
**Exposure to early life stress (ELS)**	Perceived stress (PSS[1])<br>Prenatal distress (PDQ[2])<br>Life events (LES[3])<br>Social support (Soz-U.[4])<br>Socio-demographic data<br>Maternal health risk behavior (e.g. smoking)<br>Psychosocial risks	Pre- and perinatal complications<br>Perinatal stressors (e.g. asphyxia, caesarian, preterm birth)<br>Pregnancy & obstetric history (birth weight, gestational age, birth complications)
**Maternal mental & physical health**	Maternity log data<br>semi- standardized neuropsychiatric diagnostic interview (M.I.N.I.[5])<br>Depression screening (EPDS[6])<br>Anxiety screening (STAI-S[7], STAI-T[7], ASQ[8])<br>Anthropometry<br>Individual & family history of metabolic and other medical disorders	

1, perceived stress scale; 2, prenatal distress questionnaire; 3, life experiences survey; 4, social support questionnaire; 5, Mini-international neuropsychiatric interview; 6, Edinburgh postnatal depression scale; 7, state- trait anxiety inventory; 8, anxiety screening questionnaire

Supplementary Table 1
Phenotypic characteristics of mothers and infants

Supplementary Table 1b: Summary of the information about psychopathology, socioeconomic-, psychosocial- and perceived stress of the extreme group of mothers expressed as mean ± SD1 or percentage

Variable	High prenatal ELS (n =10)	Low prenatal ELS (n =8)	*p* value
Maternal psychopathology			
**EPDS Score[3]**	15.40 ± 4.95	2.13 ± 1.25	0
**STAI-S Score[3]**	52.60 ± 13.32	32.62 ± 4.81	0.001
**STAI-T Score[3]**	50.90 ± 10.50	28.75 ± 4.53	0
**ASQ Score[3]**	5.40 ± 1.84	0.25 ± 0.46	0
**M.I.N.I. Diagnosis[4]**			
**none**	20%	100%	0
**depressive disorders**	50%	0%	0.015
**anxiety disorder**	10%	0%	ns[2]
**Current psychiatric disorder[4] (%)**			
**none**	30%	100%	0.001
**depressive disorder**	50%	0%	0.015
**anxiety disorder**	10%	0%	ns[2]
Perceived stress			
**PSS Score[3]**	32.70 ± 6.93	15.25 ± 3.92	0
**PDQ Score[3]**	23.70 ± 8.06	8.63 ± 4.37	0
Socioeconomic and psychosocial stress			
**LES-negative events Score[3]**	8.50 ± 6.98	1.63 ± 1.06	0.013
**Soz-U Score[3]**	37.80 ± 10.68	48.00 ± 6.78	0.026
**Living without a partner[4] (%)**	40%	0%	0.037
**Encouragement (Partner)[4] (%)**	70%	100%	ns[2]
**Separation(s) in the last year[4]**	50%	0%	0.015
**Daily arguments[4]**	20%	0%	ns[2]
**Physical conflicts within the preceding 12 months[4]**	60%	0%	0.005
**Composition of household >one person /room[4]**	30%	0%	0.037
**No graduation[4] (%)**	20%	0%	ns[2]
**No professional education[4] (%)**	40%	0%	0.037
**Monthly income per household ≤ 1750 Euro[4] (%)**	70%	0%	0.001
**Financial debt[4] (%)**	50%	0%	0.015

1, standard deviation; 2, not significant; 3, the first eight main variables of the principal component analysis (PCA);
4, the twelve prenatal stressors which generate the adversity score as the ninth main variable of the PCA

Supplementary Table 1
Phenotypic characteristics of mothers and infants

Supplementary Table 1c: Summary of the demographic characteristics and general medical status of mothers and infants included in the methylome analysis expressed as mean ± SD or percentage

Variable	High prenatal ELS (n = 10)	Low prenatal ELS (n = 8)	*p* value
**Maternal Age (in years)**	24.10 ± 5.43	34.00 ± 3.30	0.000
**Smoking during early pregnancy (4th to 12th wpma[1]; %)**	70%	12.50%	0.013
**Cigarettes in total (4th to 12th wpma[1])**<br>**Range of cigarettes smoked**	297.70 ± 412.68<br>0-1092	3.00 ± 8.50<br>0-24	0.050
**Smoking during late pregnancy (3rd trimester, %)**	40%	0%	0.037
**Cigarettes per day (3rd trimester)**<br>**Range of cigarettes smoked**	3.30 ± 6.31<br>0-17	0<br>0	ns[4]
**Alcohol intake during early pregnancy (4th to 12th wpma[1]; %)**	30%	75%	ns[4]
**Total alcohol intake (4th to 12th wpma[1] in g[5])**	75.50 ± 187.18	23.50 ± 22.62	ns[4]
**Alcohol during late pregnancy (3rd trimester; %)**	0%	0%	ns[4]
**Primiparous**	30%	37.50%	ns[4]
**Number of risk factors in the maternity log**	4.60 ± 2.38	3.17 ± 1.51	ns[4]
**Pre-Pregnancy BMI[2]**	25.53 ± 7.08	21.69 ± 4.57	ns[4]
**Gestational diabetes (%)**	20%	0%	ns[4]
**Gestational age at birth (wpma[1])**	38.70 ± 2.00	39.63 ± 1.60	ns[4]
**Infant's Gender (%), male**	50%	37.50%	ns[4]

1, weeks postmenstrual age; 2, body mass index; 3, standard deviation; 4, not significant; 5, gram

Supplementary Table 2

Sequences of primers used for enrichment analyses of methylated DNA and for quantitative PCR (QPCR) validation of MeDIP data.

**Human**		
Gene	Forward	Reverse
H19	5´- GAGCCGCACCAGATCTTCAG-3´	5´-TTGGTGGAACACACTGTGATCA-3´
ß-actin	5´- CCAACGCCAAAACTCTCCC-3´	5´- AGCCATAAAAGGCAACTTTCG-3´
**Monkey**		
Gene	Forward	Reverse
H19	5′-TTGGTGGAACACGCTGTGATCA-3′	5′-GAGCCGCACCAGGTCTTCAG-3′
Gapdh	5′-TTTCTTTCCTTTCGCGCTCTG-3′	5′-CCATTCATTTCCTTCCCGGTT-3′
**Rat**		
Gene	Forward	Reverse
H19	5´- CCAAGACAGAAGGGGACCAT-3´	5´- TAGATTTGGGGTTCGCCTGT-3´
ß-actin	5´- TGGGATAGTGTCCACAAGGG -3´	5´- GAAGAGTTTGGCGATGGGTG -3´
Ankyrin-3	5′- CAGGGGCTGTATTCTAGCAACT-3′	5′- CCCCTCCGTCTCACACTATTTT-3′

H19 = methylated control; *ß-actin, Gapdh* = un-methylated control

Supplementary Table 3

Characteristics of the Braincloud sample.

Sample Size	Female (Male)	Age	RIN	PMI	pH
268	92 (176)	27.9 ± 22.2	8.4 ± 0.9	26.4 ± 17.1	6.5 ± 0.3

All the data are expressed as mean ± SD

Supplementary Table 4

Characteristics of the samples used for the human behavioral and fMRI WM studies.

	Behavior	fMRI
**N**	306	174
**Gender (M/F)**	138/168	81/93
**Age**	26.0±6.2	26.9±6.4
**Handedness**	0.7±0.4	0.7±0.5
**Parental socioeconomic status**	39.9±16.5	40.9±16.4
**IQ**	107.4±11.6	109.8±11.8

Supplementary Table 5

Top diseases and biological functions associated with differentially methylated genes in the rat prefrontal cortex at PND62 after prenatal exposure to stress.

**Diseases and Disorders**	***p* value**
Neurological Disease	4.22E-09
Organismal Injury and Abnormalities	3.46E-08
Cardiovascular Disease	2.67E-07
Hereditary Disorder	4.71E-07
Psychological Disorders	4.71E-07
**Molecular and Cellular Functions**	***p* value**
Molecular Transport	2.56E-08
Cell Cycle	8.28E-08
Cell-To-Cell Signaling and Interaction	4.30E-07
Cellular Development	2.82E-06
Cellular Growth and Proliferation	5.01E-06
**Physiological System Development and Function**	***p* value**
Nervous System Development and Function	2.56E-10
Organ Morphology	1.72E-05
Embryonic Development	2.40E-05
Organ Development	2.40E-05
Organismal Development	2.40E-05

Supplementary Table 6
List of probes showing differential methylation in the eight genes resulting from the overlap of different paradigms of early life stress exposure in different species and tissues.

Gene name	Higher methylation in	Rat PFC (postnatal day 62) diff meth probe	*q* value	Monkey PFC (7 years old) diff meth probe	*q* value	Monkey CD3+ (day 30 old) diff meth probe	*q* value	Monkey CD3+ (2 years old) diff meth probe	*q* value	Human CD34+ (cord blood) diff meth probe	*q* value
Ank3	Stress	chr20:19580638-19580697	0.141959	chr9:76713019-76713067	0.143384	chr9:76897059-76897118	0.015381	chr9:76897219-76897278	0.100842	chr10:61900618-61900677	0.060659
				chr9:76713128-76713178	0.143384	chr9:76897099-76897158	0.015381	chr9:76897299-76897358	0.100842	chr10:61901941-61902000	0.185321
				chr9:76713244-76713294	0.143384	chr9:76897139-76897198	0.015381	chr9:76899099-76899158	0.017648		
				chr9:76713473-76713532	0.143384	chr9:76897179-76897238	0.015381	chr9:76899139-76899198	0.017648		
						chr9:76897299-76897358	0.015381				
						chr9:76897379-76897438	0.032022				
						chr9:77144454-77144513	0.015059				
						chr9:77144854-77144913	0.015059				
						chr9:77144934-77144993	0.015059				
	Control	chr20:19420344-19420403	0.114085	✗		✗		✗		chr10:62149544-62149598	0.146104
		chr20:19420705-19420764	0.114085							chr10:62149595-62149639	0.146104
Cnga4	Stress	chr1:163137594-163137649	0.000065	chr14:65936603-65936662	0.086013	✗		✗		chr11:6256207-6256251	0.022758
		chr1:163137327-163137372	0.000065	chr14:65936702-65936758	0.086013					chr11:6256284-6256328	0.022758
				chr14:65936991-65937039	0.086013						
				chr14:65968017-65968072	0.191803						
				chr14:65968116-65968172	0.191803						
	Control	✗		✗		chr14:65967196-65967255	0.000299	chr14:65967116-65967175	0.018034	chr11:6260165-6260210	0.095651
						chr14:65967276-65967335	0.000299	chr14:65967316-65967375	0.018034	chr11:6260273-6260320	0.095651
						chr14:65967476-65967535	0.000054	chr14:65967396-65967455	0.018034	chr11:6260342-6260386	0.095651
						chr14:65967516-65967575	0.000054	chr14:65967476-65967535	0.018034		
						chr14:65967556-65967615	0.000054	chr14:65967516-65967575	0.018034		
						chr14:65967596-65967655	0.000054				
						chr14:65967676-65967735	0.000054				
						chr14:65967716-65967775	0.000054				
						chr14:65967916-65967975	0.000054				
						chr14:65967956-65968015	0.000054				
						chr14:65968036-65968095	0.000054				
						chr14:65968076-65968135	0.000054				
Dars2	Stress	chr13:76629115-76629165	0.005713	✗		chr1:203489067-203489126	0.009011	✗		✗	
						chr1:203489147-203489206	0.009011				
						chr1:203489227-203489286	0.009011				
						chr1:203489307-203489366	0.009011				
	Control	✗		chr1:203460771-203460815	0.135128	chr1:203460535-203460594	0.016604	chr1:203460415-203460474	0.041125	chr1:173793860-173793907	0.152082
				chr1:203460900-203460956	0.135128	chr1:203460615-203460674	0.016604	chr1:203461095-203461154	0.041125		
				chr1:203460981-203461036	0.135128						
Gabrg2	Stress	chr10:27127411-27127470	0.186921	chr6:158458645-158458691	0.143637	chr6:158458012-158458071	0.056063	chr6:158457932-158457991	0.161943	chr5:161493755-161493814	0.034243
						chr6:158458052-158458111	0.056063	chr6:158458892-158458951	0.084601	chr5:161494278-161494337	0.034243
						chr6:158458412-158458471	0.001017				
						chr6:158458772-158458831	0.001017				
						chr6:158458852-158458911	0.001017				
						chr6:158458892-158458951	0.001017				
	Control	chr10:27130458-27130517	0.140810	✗		✗		✗		✗	
		chr10:27130465-27130524	0.140810								
		chr10:27130768-27130827	0.140810								
		chr10:27130966-27131025	0.140810								
		chr10:27131178-27131237	0.140810								
		chr10:27131397-27131456	0.140810								
		chr10:27104323-27104382	0.058220								
		chr10:27104987-27105046	0.164937								
Htr4	Stress	✗		✗		✗		chr6:145085774-145085833	0.132238	chr5:148034080-148034124	0.010424
								chr6:145085814-145085873	0.132238	chr5:148034081-148034125	0.010424
								chr6:145085894-145085953	0.132238	chr5:148034081-148034125	0.010424
								chr6:145163181-145163240	0.124103	chr5:148034443-148034495	0.010424
								chr6:145163261-145163320	0.124103		
	Control	chr18:58416260-58416319	0.164027	chr6:145163771-145163827	0.149301	chr6:145163981-145164040	0.100424	✗		✗	
Lphn2	Stress	✗		chr1:84116661-84116711	0.130892	chr1:84117017-84117076	0.077143	chr1:84506033-84506092	0.184338	chr1:81771325-81771384	0.176615
						chr1:84117257-84117316	0.077143	chr1:84506193-84506252	0.184338		
						chr1:84117337-84117396	0.077143	chr1:84742988-84743047	0.042337		
						chr1:84117617-84117676	0.077143	chr1:84743548-84743607	0.042337		
						chr1:84505713-84505772	0.179901				
						chr1:84505993-84506052	0.179901				
						chr1:84506033-84506092	0.179901				
						chr1:84506193-84506252	0.179901				
	Control	chr2:247201889-247201948	0.067236	✗		✗		✗		✗	
		chr2:247268119-247268163	0.012587								
Slc22a2	Stress	✗		✗		✗		chr4:157239558-157239617	0.005790	chr6:160699502-160699557	0.195967
								chr4:157240038-157240097	0.005790		
								chr4:157240078-157240137	0.005790		
	Control	chr1:42443429-42443481	0.008736	chr4:157247817-157247861	0.198596	chr4:157238718-157238777	0.024379	✗		✗	
				chr4:157248155-157248199	0.198596						
Tiam1	Stress	chr11:30218874-30218928	0.027820	✗		chr3:15243884-15243943	0.015329	✗		✗	
		chr11:30218705-30218752	0.027820			chr3:15243964-15244023	0.015329				
		chr11:30218643-30218689	0.027820								
	Control	✗		chr3:14951518-14951562	0.123568	chr3:15241884-15241943	0.064748	chr3:15242844-15242903	0.037906	chr21:32932342-32932386	0.146465
						chr3:15242084-15242143	0.064748			chr21:32932167-32932211	0.146465
						chr3:15242164-15242223	0.003634				
						chr3:15242524-15242583	0.003634				
						chr3:15242564-15242623	0.003634				
						chr3:15242644-15242703	0.003634				
						chr3:15242684-15242743	0.003634				
						chr3:15242724-15242783	0.003634				
						chr3:15242764-15242823	0.003634				

In the conditions marked with ✗, we found no probes differentially methylated between the control and stressed groups.

Supplementary Table 7

Summary of the probes associated with Ank3 and Gabrg2 which are differentially methylated between control and stressed groups.

	Higher methylation in	Rat PFC	Monkey PFC (7 years old)	Monkey CD3+ (day 30 old)	Monkey CD3+ (2 years old)	Human CD34+ (cord blood)	Monkey Whole Blood (2 years old) diff meth probe	Monkey Whole Blood (2 years old) *q* value	Monkey Buccal Cells (2 years old) diff meth probe	Monkey Buccal Cells (2 years old) *q* value
**Ank3**	Stress	✓	✓	✓	✓	✓	chr9:77074944-77075003<br>chr9:77074984-77075043	0.038705<br>0.038705	chr9:76897139-76897198	0.158048
	Control	✓	✗	✗	✗	✓	✗		✗	
**Gabrg2**	Stress	✓	✓	✓	✓	✓	✗		✗	
	Control	✓	✗	✗	✗	✗	chr6:158457292-158457351<br>chr6:158457372-158457431<br>chr6:158457852-158457911<br>chr6:158458092-158458151<br>chr6:158458132-158458191	0.001338<br>0.001338<br>0.001338<br>0.001338<br>0.001338	chr6:158457612-158457671<br>chr6:158457772-158457831<br>chr6:158458332-158458391<br>chr6:158458372-158458431	0.044732<br>0.094282<br>0.102620<br>0.102620

In the conditions marked with ✗, we found no probes differentially methylated between the control and stressed groups, while in the conditions marked with ✓ we found probes differentially methylated between the control and stressed groups.

Supplementary Table 8

List of the p values and effect size estimates ('differential') associated with probes, mapping Ank3, which were more methylated in the stressed group compared to the controls.

Samples	diff meth probe	*p* value	differential (log2)
**Rat PFC (postnatal day 62)**	chr20:19580638-19580697	0.049	0.913
**Monkey PFC (7 years old)**	chr9:76713019-76713067	0.014	1.360
	chr9:76713128-76713178	0.011	1.160
	chr9:76713244-76713294	0.037	1.090
	chr9:76713473-76713532	0.024	1.590
**Monkey CD3+ (day 30 old)**	chr9:76897059-76897118	0.010	0.966
	chr9:76897099-76897158	0.017	1.038
	chr9:76897139-76897198	0.009	1.072
	chr9:76897179-76897238	0.043	0.827
	chr9:76897299-76897358	0.024	0.871
	chr9:76897379-76897438	0.034	0.768
	chr9:77144454-77144513	0.012	0.926
	chr9:77144854-77144913	0.039	1.085
	chr9:77144934-77144993	0.022	0.948
**Monkey CD3+ (2 years old)**	chr9:76897219-76897278	0.040	0.551
	chr9:76897299-76897358	0.022	0.846
	chr9:76899099-76899158	0.047	0.562
	chr9:76899139-76899198	0.046	0.615
**Human CD34+ (cord blood)**	chr10:61900618-61900677	0.007	0.519
	chr10:61901941-61902000	0.026	0.894
**Monkey Whole Blood (2 years old)**	chr9:77074944-77075003	0.027	0.552
	chr9:77074984-77075043	0.038	1.482
**Monkey Buccal Cells (2 years old)**	chr9:76897139-76897198	0.007	1.651
